# Influence of participatory monitoring and evaluation on decision-making in maternal and newborn health programs in Mombasa County, Kenya

**DOI:** 10.1057/s41271-023-00421-w

**Published:** 2023-06-18

**Authors:** Pauline Adhiambo Oginga, Alfred Owino Odongo, Julius Njathi Nguku

**Affiliations:** 1 College of Health Sciences, School of Public Health, Mount Kenya University, Thika, Kenya; 2 Mombasa County Department of Health Services, Msanifu Kombo Road, Mama Ngina Drive, Mombasa, 81599 – 80100 Kenya

**Keywords:** Design and planning phase, Implementation phase, Initiation phase, Participatory monitoring and evaluation, Quality decision-making

## Abstract

The study assessed the utilization of participatory monitoring and evaluation approaches (PM&E) and their influence on decision-making in maternal and newborn health programs (MNH) in Mombasa County, Kenya. We conducted a cross-sectional study with a sample of 390 participants and used a structured questionnaire, modified Quality of Decision-Making Orientation Scheme, and an interview guide to collect data. We analyzed quantitative responses using descriptive statistics and binary logistic regression (at significance level of 0.05) and qualitative responses using content analysis. The study established that quality decision-making was more likely to occur in MNH programs in Mombasa County that used PM&E approaches at the initiation (OR 1.728, p < 0.05), design and planning (OR 2.977, p < 0.05), and implementation (OR 5.665, p < 0.05) phases of MNH programs than in those that did not use the PM&E approaches. This study shapes a persuasive case for improving the provision of maternal and newborn health services.

**Supplementary Information:**

The online version contains supplementary material available at 10.1057/s41271-023-00421-w.

## Key messages

- Participatory Monitoring and Evaluation is critical in collecting reliable, timely, and consistent data, which is essential for guiding stakeholders’ decision-making and actions.
- A comprehensive participatory system is important in the health sector to facilitate effective management of health systems, resource allocation, and accountability.
- Quality decision-making is more likely to occur in maternal and newborn health programs with use of participatory approaches at initiation, design and planning, and implementation phases of the programs.

## Introduction

Delivering proper maternal and newborn health programs (MNH) is a complicated process comprised of preventative, curative, and emergency elements, carried out at multiple levels of care from community to institution [1]. Efficient service delivery for mothers and newborns should be guided by participatory information collecting and sharing to guide stakeholders’ decision-making, encourage effective program implementation, and resolve emergent concerns throughout implementation [2]. In countries like Kenya, where maternal and neonatal mortality are high, participatory monitoring and evaluation systems are critical for detecting and addressing these difficulties and saving lives [3]. Participatory monitoring and evaluation involves the consideration of primary stakeholders as active participants in the initiation, design and planning, and implementation of programs; enhancing the capacity of primary stakeholders in monitoring and evaluation (to collect data, analyze, reflect and take action) through training, joint review meetings, supportive supervision and continuous mentorship; fostering joint information reviews and learning among stakeholders at various phases of the programs; and encouraging stakeholders to be accountable and responsible in taking corrective action(s) based on monitoring and evaluation findings.

The monitoring and evaluation programs enable information gathering and sharing with community stakeholders, healthcare providers, and decision-makers at the county and national levels. These programs allow varied stakeholders to influence decision-making, thus ensuring effective management of health systems, resource allocation based on need, and planned allocation of responsibility for meeting health promises [4, 5]. Participatory monitoring and evaluation programs have not been used extensively in Mombasa County due to inadequate funding for the process, lack of training for both management and implementers, lack of knowledge of its benefits, and a poor impression of the entire process by the health facilities. These problems have interfered with collection of reliable, timely, and consistent data for guiding stakeholders’ decision-making in maternal and newborn health initiatives.

Participatory techniques and stakeholder input are highly effective for tracking progress and recognizing execution issues to support planning and decision-making throughout implementation [4]. Diverse views may aid implementers and decision-makers to understand and adapt evidence to the specific context. Previous studies looked at elements of participatory monitoring and evaluation in various sectors, but little information or literature is available about this process in health programs and its role in decision-making. However, we found no study on participatory monitoring and evaluation of health programs at the county level in Kenya.

The purpose of this study was to evaluate the use of participatory methodologies and their effects on decision-making in maternal and newborn health based on self-reported assessments in a sample of 390 participants. This study focused on the extent to which Mombasa County Health Department operationalized participatory monitoring and evaluation in programs to strengthen management and to encourage a participatory form of monitoring and evaluation, including documentation of what practices the department used and, any use of it in maternal and newborn care.

## Data and methods

We conducted a descriptive cross-sectional study using a mixed methods approach, whereby we triangulated qualitative and quantitative research methodologies [6]. We obtained ethical approval from the Mount Kenya University Ethical Review Committee (approval number 1309) and research accreditation from the National Commission for Science, Technology and Innovation (license number NACOSTI/P/22/19461).

The target population was 2521 people: 1500 community health workers, 120 nurses, 570 maternity patients, 36 clinical officers in charge of health facilities, and 288 health facility management committee members, along with 7 county and sub-county reproductive health coordinators from 6 sub-counties in Mombasa County. The community health workers are volunteers, supervised by community health extension workers, who worked in 179 community health units of around 100 families or 5000 community members, which are the first tier in a four tier system of health care delivery in Kenya. All the other participants except the 7 reproductive health coordinators, whom we included as key informants, worked in 36 facilities, all levels 2 and 3 public health facilities. These facilities are in the second tier of the health care system, are under the control of the county government, and are made up of primary care health facilities with dispensaries and health centers staffed by nurses and clinical officers. The third and fourth tiers of the health care system are made up of county referral hospitals and national referral hospitals respectively.

We calculated a sample size of 345 respondents from the remaining population of 2514 using Yamane’s formula [7]:

1 $${\text{n }} = {\text{ N }} \div \, ({1 } + \, \left( {{\text{N }} \times \, 0.0{5}^{{2}} } \right),$$

where N is the population size; n is the sample size; and 0.05 is the precision rate. We then adjusted upwards to 383 respondents to accommodate a 10% probable withdrawal or non-response rate:

2 $${\text{n}}_{{1}} = {\text{ n }} \div \, \left( {{1 } - \, 0.{1}} \right),$$

where n_1_ is the adjusted sample size; n is the calculated sample size; and 0.1 is the estimated non-response rate. We then included all the 36 clinical officers in charge of the levels 2 and 3 facilities, and used proportionate stratified random sampling to sample 17 nurses, 210 community health workers, 80 maternity patients, and 40 health facility management committee members.

We then used systematic random sampling to select respondents from the respective strata to provide the members in each stratum equal opportunity to participate in the study [8]:

3 $${\text{Stratum sample size }} = \, \left( {{\text{n}}_{{1}} \div {\text{ N}}} \right) \, \times {\text{ stratum size}},$$

where N is the population size; and n_1_ is the adjusted sample size. The final sample consisted of 390 participants, including 7 key informants.

We used a structured questionnaire, a modified Quality of Decision-making Orientation Scheme **(**QoDoS) [9–13], and a Key Informant Interview (KII) guide to collect data. We attached an informed consent form to the data collection tools and respondents voluntarily completed them prior to data collection. We used a drop-off and pick-up method to administer the study questionnaire and the modified Quality of Decision-making Orientation Scheme. We used the questionnaire to assess the independent variables including; the frequency of utilization of participatory needs assessment, baseline assessments, and analysis of objectives at the initiation phase in maternal and newborn health programs (using questions 13 to 15, as listed in the Supplementary Material); the frequency of utilization of participatory feasibility analysis, Strengths, Weaknesses, Threats and Opportunities (SWOT) Analysis, and risk assessment at the design and planning phase of the programs (using questions 16 to 18, as listed in the Supplementary Material); and the frequency of utilization of participatory performance reviews, desk reviews, and supportive supervision at the implementation phase of the programs (using questions 19 to 21, as listed in the Supplementary Material).

We used the modified scheme to assess the frequency of quality decision-making practices (QDMPs) at the individual level and organizational levels of the health facilities with four indicators of quality decision-making. These include decision-making in terms of approach, culture, competence, and style [13]. These four indicators are based on 10 quality decision-making practices that this study has adopted as a catalog of ideal elements of quality decision-making at the health facilities. These elements include having a systematic, structured approach to aid decision-making (consistent, predictable and timely); assigning clear roles and responsibilities; assigning values and relative importance to decision criteria; evaluating both internal and external influences/biases; examining alternative solutions; considering uncertainty; re-evaluating as new information becomes available; performing impact analysis of the decision; ensuring transparency and provide a record trail; and effectively communicating the basis of the decision [9]. At the organizational level, we assessed the practices using two indicators of the modified scheme: decision-making approach and culture (using questions 22 to 41, as listed in the Supplementary Material). At the individual level, we evaluated the practices using the other two indicators: decision-making competence and style (using questions 42 to 65, as listed in the Supplementary Material).

### Study outcomes

The study outcomes are self-reported frequencies, for which high frequencies are perceived indications that the health facilities used participatory approaches and engaged in quality decision-making practices to a great extent. We measured the four self-reported indicators of quality decision-making using sets of 5-point Likert scales (1 to 1.8—Not at all; 1.81 to 2.6—Sometimes; 2.61 to 3.4—Frequently; 3.41 to 4.2—Often; 4.21 to 5—Always). We aggregated, using arithmetic mean, the self-reported frequencies of decision-making approach and culture, and decision making competence and style, to measure the perceived quality decision-making at the organization level and individual level respectively. We further aggregated, using arithmetic mean, the scores for quality decision making at the organization level and individual level to obtain self-reported scores of perceived quality-decision at the health facilities’ maternal and newborn programs.

We measured utilization of the nine participatory approaches, three at each program phase including initiation, design and planning, and implementation, using a 5-point Likert scale (1 to 1.8—Not at all; 1.81 to 2.6—Sometimes; 2.61 to 3.4—Frequently; 3.41 to 4.2—Often; 4.21 to 5—Always). We then aggregated, using arithmetic mean, the self-reported frequencies of the participatory approaches, each aggregated set comprising three approaches in a program phase, to measure the perceived utilization of participatory monitoring and evaluation approaches at the initiation, design and planning, and implementation phases.

We used the final aggregated self-reported scores to assess the relationship between the independent variables and the perceived quality decision making in the programs. To improve the validity of the model, we merged the five response categories in the 5-point Likert scale to obtain two response categories, ‘rarely’ and ‘often’. To determine the category intervals, we subtracted the lowest point in the 5-point Likert scale from the highest point and divided the difference by the required number of categories [(5–1)/2]. Therefore, we recoded the aggregated self-reported scores ranging between 1 and 3 to a response category named ‘rarely’ and recoded scores ranging between 3.1 and 5 to a response category named ‘often’. The category ‘rarely’ signified that the extent of use or practice was minimal while the category ‘often’ signified that the extent of use or practice was great.

We performed descriptive analyses (using arithmetic mean, standard deviation and coefficient of variation) to summarize data on participants' demographics and specific variables and binary regression analysis (at a significance level of 0.05) to detect relationships between the selected frequency in individual questions and variables and the dependent variable. We conducted a Phi coefficient test, at a significance level of 0.05, to determine the strength of association between the frequency of quality decision making practices (dependent variable) and the independent variables, such as the frequency of utilization of participatory monitoring and evaluation approaches at the initiation phase, frequency of utilization of participatory monitoring and evaluation approaches at the design and planning phase, and frequency of utilization of participatory monitoring and evaluation approaches at the implementation phase. The Phi coefficient ranges from − 1 to + 1 with a negative coefficient signifying negative relationship, zero signifying no relationship, and a positive coefficient signifying positive relationship between the dependent variable and an independent variable. A Phi coefficient greater or less than zero with a p-value (significance level) less than 0.05 was deemed to indicate significant association between an independent variable and the outcome variable.

We used a binary logistic regression analysis to determine the perceived degree of influence of participatory monitoring and evaluation approaches at the initiation, design and planning, and implementation phases on self-reported frequency of decision-making (at 0.05 significance level). We set the last response category as the reference group. The Hosmer–Lemeshow test yielded a significance value greater than 0.05 indicating that the model adequately fit the data: that is, there was no difference between the observed and predicted models. The results also indicated that the model correctly classified 65.3% of cases. The Nagelkerke R2 test indicated that the model (utilization at the initiation, design and planning, and implementation phases) explained 20.9% of the variance in quality of decision-making. An odds ratio of 1.0 indicated that an independent variable was not associated with quality decision-making (dependent variable). An odds ratio of greater than 1.0 indicated that the independent variable was a catalyst for quality decision-making practices. An odds ratio of less than 1.0 indicated that the independent variable was an inhibitor of quality decision-making practices. An odds ratio greater or less than 1.0 with a p-value (significance level) less than 0.05 was deemed to indicate significant influence of the independent variable on the outcome variable.

We conducted the key informant interviews on the same day and recorded all sessions using a digital voice recorder, then transcribed them verbatim. Each interview session lasted about 60 min. To assess qualitative data from the interviews we used content analysis. The IBM statistical package for the social sciences (for Windows), version 25, was used for quantitative analysis, while content analysis was conducted manually.

## Results

### Quality of decision-making

Findings indicated that decision-making practices at the health facilities perceived to constitute ideal approach to decision-making (Table 1, 1) as well as ideal competencies to decision-making (Table 1, 3) were more frequently reported by the participants. Findings also indicated that decision-making practices at the health facilities perceived to constitute ideal culture to decision-making (Table 1, 2) and ideal style of decision-making (Table 1, 4) were less frequently reported by the participants. Furthermore, findings of the aggregated scores for the two indicators, approach and culture, indicated that decision-making practices at the organization level perceived to constitute quality decision-making were frequently reported by the participants (Table 1, 5). Additionally, findings of the aggregated scores for the other two indicators, competence and style, indicated that decision-making practices at the individual level perceived to constitute quality decision making were frequently reported by the participants (Table 1, 6). The individual scores did not vary significantly from the average scores (CV < 30%) indicating that the mean score were representative of the respondent’s opinion.

**Table 1:** Average reported frequencies

No.	Decision-making practices	Level	Mean	SD	CV (%)	Frequency interpretation
1	Perceived ideal approach to decision making	Organization level	3.89	0.321	8	Often
2	Perceived ideal culture to decision making	Organization level	2.05	0.485	24	Sometimes
3	Perceived ideal competencies in decision making	Individual level	3.75	0.454	12	Often
4	Perceived ideal style of decision making	Individual level	1.99	0.273	14	Sometimes
5	Perceived quality of decision making at the organization level		3.00	0.240	7	Frequently
6	Perceived quality of decision making at the individual level		2.94	0.287	10	Frequently
7	Perceived quality decision making in maternal and newborn health programs		2.95	0.223	8	Frequently

Findings of the aggregated scores for decision-making practices at both organization level and individual level indicated that decision-making practices at the health facilities perceived to constitute quality decision making were frequently reported by the participants (Table 1, 7). The individual scores did not vary significantly from the average score (CV < 30%) indicating that the mean score was representative of the respondent’s opinion.

### Approaches in the initiation phase

The respondents indicated that at the programs’ initiation phase, health facilities often conducted participatory analysis of objectives (${\overline{\text{X}}}$  = 3.50, σ = 0.628, CV = 18%). The respondents’ opinions diverged (CV > 30%) on the frequency with which the health facilities conducted participatory baseline assessment (${\overline{\text{X}}}$ = 2.96, σ = 0.901, CV = 30%) and participatory needs assessment (${\overline{\text{X}}}$ = 2.24, σ = 0.931, CV = 42%). Furthermore, findings of the aggregated scores for the three indicators showed that participants perceived that the health facilities frequently utilized participatory monitoring and evaluation approaches at the programs’ initiation phase (${\overline{\text{X}}}$ = 2.91, σ = 0.648, CV = 22%). Findings from the key informant interviews also showed that health facilities used participatory approaches at the initiation phase of the programs in Mombasa County (Table 2, KII 6).

**Table 2:** Key informant responses

ID	Response
KII 6	“…Under the youth friendly sexual and reproductive health initiative in the county, focus group discussions with the youth are conducted within youth friendly clinics, to understand the sexual and reproductive health needs of the youth from their own perspective rather than from the providers’ view point, in order to come up with services that respond to their needs. This approach has helped in increasing the number of youth seeking sexual and reproductive health services in the youth friendly clinics…”
KII 4	“Yes, we use SWOT analysis in a collaborative and participatory manner in the development of plans to define the framework of the MNH programs’ main threats associated with weaknesses and significant opportunities linked to our strengths….”
KII 2	“We organize for Workshops where we discuss various plans before they are implemented…some of the activities that is done during the planning workshops include feasibility analysis, risk assessment, budget analysis etc.….Staff from different departments are involved…a number of stakeholders, especially members of the health facility management committees, are involved in the workshops.”
KII 1	“We conduct supportive supervision visits to the health facilities on a quarterly basis….Supportive supervision visits involve multiple activities, such as observation of all departments, review of registers and reports, training, and follow up on issues identified in the previous visit; however, the majority of the visit is dedicated to data checking.”
KII 6.1	“Supportive supervision starts from planning… when we plan we are building the capacity of the supervising teams. So, to do this we plan together, we prepare for supportive supervision logistically, manpower, checklists, and so forth.”

### Design and planning phases

The respondents indicated that health facilities often conducted participatory feasibility analysis (${\overline{\text{X}}}$ = 3.50, σ = 0.628, CV = 18%) and participatory SWOT analysis (${\overline{\text{X}}}$ = 3.88, σ = 0.458, CV = 12%). For participatory risk assessment, use was frequent (${\overline{\text{X}}}$ = 3.06, σ = 0.798, CV = 26%). Responses to all the statements yielded CV < 30% showing that the mean aggregates represented the collective opinions of the respondents. Additionally, findings of the aggregated scores for the three indicators revealed that participants perceived more frequent utilization, by health facilities, of participatory monitoring and evaluation approaches at the programs’ initiation phase (${\overline{\text{X}}}$ = 3.59, σ = 0.598, CV = 17%). Findings from the interviews also revealed use of participatory approaches at the design and planning phases of the programs studied in Mombasa County (Table 2, KII 4 and KII 2).

### Implementation phase

The respondents indicated that supportive supervision (${\overline{\text{X}}}$ = 3.95, σ = 0.356, CV = 9%), participatory performance reviews (${\overline{\text{X}}}$ = 3.81, σ = 0.581, CV = 15%) and participatory desk reviews (${\overline{\text{X}}}$ = 3.96, σ = 0.373, CV = 9%) were often conducted by the health facilities. Responses to all the statements yielded CV < 30% showing that the mean aggregates represented the collective opinions of the respondents. Moreover, findings of the aggregated scores for the three indicators revealed that participants perceived more frequent utilization, by health facilities, of participatory monitoring and evaluation approaches at the programs’ initiation phase (${\overline{\text{X}}}$ = 3.80, σ = 0.503, CV = 13%). Results from the interviews also revealed use of participatory approaches at the implementation phase (Table 2, KII 1 and KII 6.1).

### Association between participatory approaches and quality of decision-making

The results indicated that PM&E approaches at the initiation phase (ϕ = 0.164, p < 0.05), at the design and planning phase (ϕ = 0.203, p > 0.05), and at the implementation phase (ϕ = 0.199, p < 0.05) had a weak positive relationship with quality decision-making (Table 3).

**Table 3:** Association between utilization of PM&E approaches and quality of decision-making

Independent variable	Frequency	Quality decision-making		Phi coefficient		
		Not improved	Improved	Value	N	Sig.
Initiation phase	Rarely	117	136	0.164	349	0.002
	Often	27	69			
Design and planning phase	Rarely	26	11	0.203	349	0.000
	Often	118	194			
Implementation phase	Rarely	15	3	0.199	349	0.000
	Often	129	202			

Influence of participatory approaches on Quality of Decision-Making

The odds of a health facility making quality decisions in maternal and newborn health programs were 1.73 times higher when they used participatory approaches at the initiation phase than when facilities used them rarely, with a 95% CI of 1.02 to 2.92. The results also showed that the odds of a health facility making quality decisions were 2.98 times higher when they used these participatory approaches at the design and planning phase than when facilities rarely used them, with a 95% CI of 1.38 to 6.42. The odds of a health facility making quality decisions in maternal and newborn health programs were 5.67 times higher with use of participatory approaches at the implementation phase than when facilities rarely utilized them, with a 95% CI of 1.57 to 20.46 (Table 4).

**Table 4:** Influence of utilization of PM&E approaches on quality of decision-making

	B	S.E.	Wald	df	Sig.	Exp(B)	95% CI for Exp(B)	
							Lower	Upper
Initiation phase	0.547	0.267	4.193	1	0.041	1.728	1.024	2.917
Design and planning phase	1.091	0.392	7.730	1	0.005	2.977	1.380	6.424
Implementation phase	1.734	0.655	7.007	1	0.008	5.665	1.569	20.459
Constant	− 2.421	0.716	11.422	1	0.001	0.089		

a. Variable(s) entered on step 1: PM&E at the Initiation phase, PM&E at the Design & Planning Phase, and PM&E at the Implementation Phase

## Discussion

The results indicated that decision-making practices at both the individual level and organization levels in maternal and newborn health programs in Mombasa County were generally favorable in terms of Quality Decision-Making Practices (QDMPs). A few practices needed improvement, including provision of training for stakeholders in the science of decision-making. This is essential for providing and enhancing the management and leadership skills for every stage of the programs and for achieving goals and generating results needed. Kananura et al. identified the need to improve PM&E skills expand the spaces for decision-making for both key implementers and decision-makers [4].

The findings that the health facilities frequently used participatory approaches at the initiation phase are inconsistent with the findings of Sifunjo who found that stakeholders in maternal and newborn health programs in Kajiado County were highly involved in needs assessment, project identification, and objective analysis among other participatory approaches at the initiation phase [14]. Because we found that participatory monitoring and evaluation approaches at the initiation phase of the programs was a significant predictor of quality decision-making, health facilities that utilize participatory monitoring and evaluation approaches at the initiation phase of maternal and newborn health programs have a high likelihood of making quality decisions. This is supported by the findings of Sifunjo who found that participatory project identification resulted in the long-term viability of maternal health programs [14]. That study also found that stakeholder engagement in vision, mission, and target setting improved the success of maternal health programs.

Our findings that participatory approaches at the design and planning phase were more frequently utilized are consistent with the findings of Kananura et al.[4]. Their study established that during the design phase of the maternal and newborn program in eastern Uganda, district level health officers conducted focus group discussions and stakeholder meetings with local community members to identify local problems, feasible solutions, and local resources that would support the program. These included infrastructure and governance structures, human and financial resources. The results are also consistent with the findings of Kajaga [15]. That study established that stakeholders in a United States Development Agency, Supporting an AIDS-Free Era (USAID-SAFE) program in Gulu District of Uganda engaged in the design and planning phase. Intended beneficiaries took part in planning, in setting indicators, priorities, targets, and objectives, in problem identification, and in proposing solutions to challenges. These findings differ from those of Karanja [16]. The latter revealed that stakeholders were not engaged during the panning phase of Constituency Development Fund projects in Dagoretti South Sub-County, Kenya. The results by Kajaga also showed utilization of participatory approaches at the design and planning phase of maternal and newborn health programs to have been a significant predictor of quality decision-making. Hence, health facilities that utilize participatory approaches at the design and planning phase of maternal and newborn health programs have higher probability of making quality decisions. The study of Kananura et al. supports this [4].

We found participatory approaches at the implementation phase to have been highly utilized. This is consistent with Kananura et al. who found facilities they studied supported supervision and mentorship visits, quarterly review meetings and midterm surveys with various key stakeholders, including community members, health workers, and sub-county and district health teams [4]. Our results are also consistent with the findings of Kajaga [15] whose study showed use of participatory approaches to a great extent during the implementation phase of a program sponsored by the United States aid agency, called Supporting an AIDS-Free Era (SAFE) program, in Gulu District, Uganda. Those findings differ from Karanja’s who found that stakeholders had not engaged in the implementation phase of Constituency Development Fund projects in Dagoretti South Sub-County, Kenya [16]. The findings by Karanja further showed that participatory approaches at the implementation phase of maternal and newborn health programs had been a significant predictor of quality decision-making. Thus, health facilities that utilize PM&E approaches for maternal and newborn care at the implementation phase have a high chance of making quality decisions. Again, this is consistent with Kananura et al. who showed that at the implementation phase, the PM&E approaches provided factual data that formed the basis for decision-making and assisted in the identification of evolving issues [4].

### Study limitations

The presented observations were derived from self-assessment, self-rating and thus represent perceived effects, and not a randomized trial where health facilities with and without monitoring and evaluation program were compared in terms of objective measures of efficacy and efficiency. This study relies on the associations between the self-reported frequency of using participatory monitoring and evaluation approaches and the frequency of perceived quality decision-making practices in maternal and newborn health programs. We recommend the need for hypothesis driven studies with proper design to strengthen or refute our conclusions.

## Conclusions

We conclude that in the opinion of select participants, utilization of participatory monitoring and evaluation approaches at the initiation, design and planning, and implementation phases had a significant effect on quality of decision-making in maternal and newborn health programs in Mombasa County. Quality decision-making was more likely to occur with utilization of participatory monitoring and evaluation approaches at the initiation, design and planning, and implementation phases than without its use. This study shapes a persuasive case to government and non-governmental public health actors that active stakeholder participation in undertaking monitoring and evaluation of maternal and newborn health programs is a critical precursor for quality decision making in the programs. These findings are valuable to maternal and newborn health program managers, decision makers and implementers in the County Government of Mombasa and various health facilities within Mombasa County in providing critical information for evidence-based policy development to support and sustain participatory monitoring and evaluation towards enhancing quality decision-making in the provision of maternal and newborn health services.

## Supplementary Information

Below is the link to the electronic supplementary material.

Supplementary file1 (PDF 2115 KB)
