# Supplementary material for: Influence of participatory monitoring and evaluation on decision-making in maternal and newborn health programs in Mombasa County, Kenya
Source: J Public Health Policy. 2023 Jun 18;44(3):435–48. doi: 10.1057/s41271-023-00421-w (PMC10485134; doi:10.1057/s41271-023-00421-w)
Supplement: Supplementary file 1 — Supplementary file1 (PDF 2115 KB) [file 41271_2023_421_MOESM1_ESM.pdf]

INFLUENCE OF PARTICIPATORY MONITORING AND EVALUATION ON DECISION-MAKING IN MATERNAL AND NEWBORN HEALTH PROGRAMS IN MOMBASA COUNTY, KENYA

PAULINE ADHIAMBO OGINGA

A THESIS SUBMITTED IN PARTIAL FULFILMENT OF THE REQUIREMENT FOR THE AWARD OF MASTERS DEGREE IN PUBLIC HEALTH OF MOUNT KENYA UNIVERSITY

MAY 2023

DECLARATION AND APPROVAL

Declaration

This Thesis is my own work, and it has not been submitted for a degree or any other award at any other University.

Signed:.. Date: 2nd May 2023

Pauline Adhiambo Oginga

MPH/2018/39091

Approval

We affirm that the candidate completed the work described in this thesis while under our supervision.

Signed:.. Date: 17th May 2023

Dr. Alfred Owino Odongo, PhD

School of Public Health

Mount Kenya University

Signed:.. Date: 17th May 2023

Dr. Julius Njathi Nguku, PhD

School of Public Health

Mount Kenya University

DEDICATION

I dedicate this work to my family for their love and support during its conceptualization and execution.

ACKNOWLEDGEMENTS

I thank the Almighty for his bountiful Grace, generosity, and genuine favor in my studies. I applaud the MKU School of Public Health for creating a welcoming learning atmosphere. I am grateful to my supervisors, Dr. Alfred Owino and Dr. Julius Nguku, for their recommendations, critiques, and remarks, which have helped shape my research. I'd want to offer my appreciation to my classmates for their academic and spiritual support during the course.

ABSTRACT

Improving Maternal and Newborn Health (MNH) is crucial to attaining the SDGs and Kenya Vision 2030. Both demand and supply-side issues exist in environments where maternal and neonatal mortality is high. Participatory Monitoring and Evaluation (PM&E) systems are critical for recognizing these difficulties, which may then be minimized to save lives. Participatory M&E of health programs enables information collecting and sharing with community stakeholders, service providers, and county and national decision makers. As a result, this impacts decision-making and action by numerous stakeholders in order to achieve community emancipation, effective administration of health systems, resource allocation based on need, and responsibility for meeting health obligations. This study assessed the utilization of PM&E approaches and its influence in decision making in MNH programs in Mombasa County. The specific objectives included determining the influence of utilization of PM&E approaches at the program initiation phase on the quality of decision making in MNH programs in Mombasa County; assessing the influence of utilization of PM&E approaches at the program design and planning phase on the quality of decision making in MNH programs in Mombasa County; and evaluating the influence of utilization of PM&E approaches at the program implementation phase on the quality of decision making in MNH programs in Mombasa County. A descriptive cross-sectional study was conducted using mixed methods research approach. The research was conducted at Mombasa County's 36 level 2 and 3 public health facilities. Purposive and stratified random sampling techniques were used to select 390 participants from a target population of 2521 county and sub-county reproductive health coordinators, nurses, community health workers, maternity patients, facility in-charges, and members of health facility management committees. Data was gathered from 349 survey respondents and 7 interviewees through the use of a structured questionnaire, modified Quality of Decision-Making Orientation Scheme (QoDoS), and key informant interviews, and descriptive and inferential statistics were used to analyze the data. At a significance level of 0.05, associations between variables were determined using the phi correlation coefficient and binary logistic regression. The results revealed that the odds of quality decision making occurring were higher when PM&E approaches were utilized at the initiation ($\phi = 0.164$, $OR = 1.728$, $p < 0.05$), design and planning ($\phi = 0.203$, $OR = 2.977$, $p < 0.05$), and implementation ($\phi = 0.199$, $OR = 5.665$, $p < 0.05$) phases of MNH programs than when the PM&E approaches were not utilized. The study concludes that utilization of PM&E approaches had a significant effect on quality of decision making in MNH programs in Mombasa County. On the basis of the research findings, I recommend that the department of health in Mombasa County should adopt PM&E approaches in MNH programs' initiation, design and planning, and implementation phases in order to benefit from rational, timely, and relevant information for decision-making, and thus improve on delivery of MNH services.

TABLE OF CONTENTS

DECLARATION AND APPROVAL ii

DEDICATION iii

ACKNOWLEDGEMENTS iv

ABSTRACT v

TABLE OF CONTENTS vi

LIST OF TABLES ix

LIST OF FIGURES x

LIST OF ABBREVIATIONS AND ACRONYMS xi

CHAPTER ONE: INTRODUCTION 1

1.1 Background of the Study 1

1.2 Statement of the Problem 8

1.3 Objectives of the Study 10

1.4 Research Questions 10

1.5 Significance of the Study 11

1.6 Scope of the Study 12

1.7 Limitations of the Study 12

1.8 Delimitations of the Study 13

1.9 Assumptions of the Study 13

1.10 Operational Definition of Key Terms 14

CHAPTER TWO: LITERATURE REVIEW 16

2.1 Introduction 16

2.2 Empirical Literature 16

2.3 Research Gaps 18

2.4 Participatory Monitoring and Evaluation 20

2.5 Participatory M&E and Decision Making in MNH 25

2.6 Theoretical Framework 28

2.7 Conceptual Framework 31

2.8 Recap of Literature Review 33

CHAPTER THREE: RESEARCH METHODOLOGY 35

3.1 Introduction 35

3.2 Research Methodology 35

3.3 Research Design 35

3.4 Location of the Study 36
3.5 Target Population 37
3.6 Sampling Procedures and Techniques 39
3.7 Sample Size 40
3.8 Construction of Research Instruments 41
3.9 Pre-testing, Validity and Reliability 42
3.10 Data Collection Methods and Procedures 44
3.11 Data Analysis Techniques and Procedures 45
3.12 Ethical Considerations 45
CHAPTER FOUR: RESULTS AND DISCUSSIONS 48
4.1 Introduction 48
4.1.1 Response Rate 48
4.1.2 Reliability of Results 48
4.2 Descriptive Results 49
4.2.1 Socio-Demographic Information 49
4.2.2 Utilization of PM&E in MNH Programs 52
4.2.3 Quality of Decision Making in MNH Programs 54
4.2.4 Utilization of PM&E Approaches at the MNH Programs' Initiation Phase 56
4.2.5 Utilization of PM&E at the Design and Planning Phase in MNH Programs 58
4.2.6 Utilization of PM&E at the Implementation Phase in MNH Programs 60
4.3 Correlation Analysis 62
4.4 Influence of Utilization of PM&E Approaches on Decision-Making 64
CHAPTER FIVE 68
SUMMARY OF FINDINGS, CONCLUSIONS AND RECOMMENDATIONS 68
5.1 Introduction 68
5.2 Summary of Findings 68
5.3 Conclusions 70
5.4 Recommendations 70
5.5 Suggestions for Further Research 71
REFERENCES 72
APPENDICES 78
Appendix 1: Participant's Consent Form 78
Appendix 2: Research Questionnaire 81

Appendix 3: Interview Guide....................88
Appendix 4: Map of Mombasa County....................92
Appendix 5: Distribution of Health Facilities in Mombasa County....................93
Appendix 6: Ethical Approval....................94
Appendix 7: NACOSTI Research Permit....................95
Appendix 8: Similarity Index Report....................96

LIST OF TABLES

Table 2.1: Research Gaps 18
Table 2.2: Operationalization of Variables 32
Table 3.1: Target Population 38
Table 3.2: Distribution of the Sample 41
Table 4.1: Questionnaire Response Rate 48
Table 4.2: Reliability Tests 49
Table 4.3: Socio-Demographic Information 50
Table 4.4: Utilization of PM&E in MNH Programs in Mombasa County 52
Table 4.5: Quality of Decision Making in MNH Programs in Mombasa County 56
Table 4.6: Utilization of PM&E Approaches at the Initiation Phase in MNH Programs in Mombasa County 57
Table 4.7: Utilization of PM&E Approaches at the Design & Planning Phase in MNH Programs in Mombasa County 59
Table 4.8: Utilization of PM&E Approaches at the Implementation Phase in MNH Programs in Mombasa County 61
Table 4.9: Phi Correlation Coefficient 63
Table 4.10: Hosmer and Lemeshow Test 64
Table 4.11: Contingency Table for Hosmer-Lemeshow Test 64
Table 4.12: Classification Table 65
Table 4.13: Model Summary 65
Table 4.14: Binary Logistic Regression 67

LIST OF FIGURES

Figure 2.1: The 10 QDMPs 28

Figure 2.2: Conceptual Framework 33

LIST OF ABBREVIATIONS AND ACRONYMS

**CDF**	Community Development Fund
**CHEWs**	Community Health Extension Workers
**CHSIP**	County Health Strategic and Investment Plan
**CHWs**	Community Health Workers
**FGDs**	Focus Group Discussions
**FPR**	Farming Participatory Research
**GOs**	Government Organizations
**IBM**	International Business Machines Corporation
**IERC**	Institutional Ethical Review Committee
**IMR**	Infant Mortality Rate
**KIIs**	Key Informant Interviews
**LASDAP**	Local Authority Service Delivery Action Planning
**M&E**	Monitoring and Evaluation
**MMR**	Maternal Mortality Rate
**MNCH**	Maternal, Neonatal and Child Health
**MNH**	Maternal and Newborn Health
**MOH**	Ministry of Health
**MPCU**	Municipal Planning and Coordinating Unit
**NACOSTI**	National Commission for Science, Technology and Innovation
**NGOs**	Non-Governmental Organizations
**NMR**	Neonatal Mortality Rate
**OECD**	Organization for Economic Cooperation and Development
**PAR**	Participatory Action Research
**PM&E**	Participatory Monitoring and Evaluation

**PRA**	Participatory Rural Appraisal
**qual**	Qualitative
**QUAN**	Quantitative
**SDGs**	Sustainable Development Goals
**SPSS**	Statistical Package for Social Sciences
**UNICEF**	United Nations Children Fund
**USAID**	United States Agency for International Development
**SAFE**	Supporting an AIDS-Free Era
**WHO**	World Health Organization

CHAPTER ONE: INTRODUCTION

1.1 Background of the Study

1.1.1 History of Participatory Monitoring and Evaluation (PM&E)

Documented PM&E experiences date back to the 1970s and draw on a 40-year tradition of participatory research, including Farming Participatory Research (FPR) or Farming Systems Research (FSR), Participatory Rural Appraisal (PRA), Participatory Action Research (PAR), and Participatory Learning and Action (PLA) (Lubita, Mubati & Mulonda, 2017). As PM&E became increasingly frequently employed in the 1980s, particularly in the policymaking arena, practitioners saw it solely as a technical application of a "toolbox" of techniques, at the detriment of individual and community empowerment (Sifunjo, 2019). According to Jackson and Kassam, as reported by Onyango (2018), the 1990s witnessed a re-conceptualization of PM&E, particularly with a focus from major donor organizations to engage additional stakeholders participating in development interventions, in response to a plea for more recognition and precision in analyzing stakeholder participation. As a developing discipline, PM&E has been progressively implemented in many settings and hundreds of development programs throughout the world, gaining diverse roles as individuals and organizations acquire more knowledge on how to acclimatize, transform, and experiment with participatory ways (Onyango, 2018).

1.1.2 Definition of PM&E

Understanding PM&E is heavily reliant on how participation is perceived. Participation may be described as the procedure for including and influencing stakeholders in development decision-making, resource allocation, implementation, and control (Mubita et al., 2017). Participation stresses the development of structures and procedures that incorporate people most directly impacted by the program, as well as those who are typically powerless and/or voiceless in the design and execution of the program (Rossman, 2015). Specific attention has

grown in PM&E to challenge more typical top-down to bottom-up assessment methodologies. The PM&E concept is based on recognizing the limitations of traditional M&E and focuses on deepening participation. It is a process that goes hand in hand with learning and empowerment, determining how progress should be made by engaging indigenous residents, policy makers and development agencies in shared decision-making and acting on the results (Onyango, 2018). Participatory M&E involves main stakeholders in decision making by allowing them to drive the M&E process, improve their abilities, and, when necessary, support them in making their own decisions on problems that impact them (Kananura et al., 2017).

The PM&E process is therefore focused on identifying how to evaluate progress, agreeing on expected outcomes and milestones, acquiring appropriate data, performing collaborative analysis, and deciding on actions (Hilhorst & Guijt, 2006). As a result, the primary phases in putting PM&E into reality are: planning a PM&E process; data collection and analysis; and documenting, reporting, and information sharing. During the planning phase, the stakeholder groups are identified and chosen; PM&E problems, needs, and expectations are identified; and PM&E objectives and indicators are defined. Following the identification of PM&E needs and objectives, the next important phase in the PM&E process is collecting and gathering information. After data collection, the subsequent step of PM&E comprises processing or analyzing gathered data, while ideally, analysis should occur throughout the PM&E cycle (Bell & Aggleton, 2016). The final step of PM&E comprises documenting, reporting, and sharing of findings created by the PM&E process, as well as relating them to action. The outcomes of M&E operations are shared with other stakeholders in this stage, and appropriate actions are deliberated based on the outcomes (Onyango, 2018).

The planning stage is often regarded as the most fundamental to the success and efficacy of the PM&E process. At the outset, it is necessary to identify and choose the stakeholder groups

that will be participating in the PM&E process planning. These are those who have an impact on or are impacted by a development program and have fundamental rights as citizens to voice their views on public problems. It is critical to construct structures that enable engagement with interested parties and the creation of a diverse variety of participatory procedures (Mohammed et al., 2018). After the identification and selection of stakeholders, defining the PM&E project's objectives is crucial. This approach should include extensive talks amongst stakeholders about the various parties' requirements and aims (Luutu, 2016). To identify the objectives of PM&E, one must first understand who the participants in the process are, as well as who the end-users will be, why the project is being carried out, and how the outcomes and process will be used. After agreeing on PM&E objectives, indicators may be created. In general, indicators are distinct from objectives in that indicators are considered to be relatively concrete and particular, but objectives may represent the project's or initiative's larger overall viewpoints, representing its values, ambitions, and long-term aims (Bell & Aggleton, 2016). The choice of indicators will ultimately be determined by what is being assessed, who the end users are, and how the information will be used.

Following the identification of information needs and objectives, the next important phase in the PM&E process is selecting how to collect and gather information. A wide range of methods and approaches can be employed to collect data. The selection of tools will be heavily influenced by the environment and project-specific requirements. Several critical issues must be addressed at this point, including where the information will be collected, which technologies should be utilized, and who will acquire the information and when (Bell & Aggleton, 2016). Following data collection, the subsequent step of PM&E comprises processing or analyzing gathered data, while ideally, analysis should occur throughout the PM&E cycle (Bell & Aggleton, 2016). Defining the scope of data analysis, particularly what is to be studied, how, and by whom, is an important part of creating PM&E. (Rossman, 2015).

The methods or procedures used to collect data, as well as the information requested by participants, will determine how data is analyzed (Glandon et al., 2017).

The final step of PM&E comprises documenting, reporting, and sharing of findings created by the PM&E process, as well as relating them to action. Participatory M&E is fundamentally about information exchange amongst program recipients, program executors, donors, and, in certain instances, external evaluation experts (Rossman, 2015). Records of acquired data are presumably retained from the beginning of the project in order to give a 'holistic' picture during the course of the endeavor. Regardless of how participatory the M&E process has been in the preceding processes, not all stakeholders can be included in M&E data gathering and analysis. The outcomes of M&E operations are shared with other stakeholders in this stage, and appropriate actions are deliberated based on the outcomes (Onyango, 2018).

As a consequence, PM&E is a self-assessment, collective knowledge creation, and collaborative work process in which program or intervention stakeholders collaborate to identify evaluation problems, gather and analyze data, learn from others, and act (Onyango, 2018). Participatory M&E attempts to boost key stakeholders' participation as active participants in interventions by measuring and assessing progress toward collectively agreed-upon outcomes, taking the initiative in decision-making, and taking remedial action (Kananura et al., 2017). When programs and organizations have strong communication and feedback loops in place, this strategy contributes to needs-based planning, decision-making, and better accountability. PM&E enables individuals directly impacted to draw on both failures and triumphs by including stakeholders in decision-making, resource allocation, execution, and administration of development efforts, as well as delegating responsibility to people (Rossman 2015).

1.1.3 Benefits of the PM&E approach

The PM&E strategy has several advantages, including providing management and stakeholders with reasonable, timely, and relevant information for decision-making, improving performance, and empowering beneficiaries to take ownership of the projects (Kathongo, 2018). Participatory M&E is not an objective in itself; rather, it may be used as a management tool to manage resources, social interactions within a certain region, or between local people and outside organizations. The primary goal of PM&E is to provide data to beneficiaries and program managers in order for them to review if project objectives were met and how the appropriate authorities used resources to improve project management and make key choices (Karanja, 2016).

The health-care system has grown increasingly conscious of the significance of including communities and users in the administration of health-care institutions and the evaluation of service delivery. One option is the formation of 'health facility management committees,' which are charged with garnering local support, mobilizing funding for operating expenditures and upkeep, and establishing relationships with communities and end-users (WHO, OECD & World Bank, 2018; Waweru et al., 2013). In Kenya, for example, level 2 and 3 public health institutions are supervised by Facility Management Committees. Each public primary health care facility has a health facility management committee comprised of nine (9) elected members. These committees' membership includes the facility in-charge (who serves as the committee's secretary and is ex-officio), an area Member of County Assembly, a ward administrator, a religious leader, a youth representative, a community representative, a business representative, a women representative, and a disability representative. The committee meets regularly and is in charge of mobilizing resources, approving facility budgets and procurement strategies, and ensuring that community requirements are addressed.

Furthermore, these committees must actively monitor and evaluate the effectiveness, efficiency, and equality of service delivery.

A comprehensive PM&E system is a valuable tool in the healthcare industry because it allows decision-makers, budget planners, program implementers (service providers), and community stakeholders (such as households, community local leaders, and community health workers) to figure out which strategies work and which ones need to be tweaked to facilitate effective management of health systems, resource allocation based on need, and taking responsibility for keeping health-care promises (Kananura et al., 2017; Holvoet & Inberg, 2014). For example, providing adequate MNH is a complex process involving a variety of preventative, curative, and emergency treatments, including multiple levels of care ranging from the community to the institution and beyond. As a result, efficient MNH service delivery should be guided by participatory information collecting and sharing in order to guide stakeholders' decision-making, encourage effective program implementation, and resolve emergent concerns throughout implementation. According to Kananura et al., (2017), at the design stage, PM&E methodologies are effective for identifying major local challenges and potential local solutions, as well as informing succeeding efforts. During the implementation phase, PM&E methodologies give evidence that informs decision-making and aids in the identification of emergent concerns, such as poor execution by certain health teams and health facility limits. When this information is shared with concerned stakeholders, it pushes them to take necessary action.

1.1.4 Health System Infrastructure and PM&E in Mombasa County

In compliance with standards and laws, the Mombasa county health delivery system is organized into four levels of care. The four stages are community, primary care, primary referral, and secondary referral. Community services seek to build service demand, whereas primary care and referral services seek to meet that demand. The county is home to the Coast

Level Five Hospital, a referral facility that serves the whole coast region. Port Reitz, Tudor, Likoni, and Kenya Navy are all level four (4) public hospitals. There are 11 health clinics and 25 public dispensaries (Mombasa County Government, 2018). These are supplemented by other notable private hospitals.

Levels 2 and 3 public health institutions are supervised by Facility Management Committees. Each public primary health care facility has a health facility management committee comprised of nine (9) elected members. These committees' membership includes the facility in-charge (who serves as the committee's secretary and is ex-officio), an area Member of County Assembly, a ward administrator, a religious leader, a youth representative, a community representative, a business representative, a women representative, and a disability representative. The committee meets regularly and is in charge of mobilizing resources, approving facility budgets and procurement strategies, and ensuring that community requirements are addressed. Furthermore, these committees must actively monitor and evaluate the effectiveness, efficiency, and equality of service delivery.

PM&E is not widely practiced in Mombasa County, as it is in other Kenyan counties, due to a number of factors, including inadequate funding for the PM&E process, a lack of knowledge of the need for PM&E, a poor opinion of the overall PM&E process, and a lack of PM&E training for both county administration and implementers (Jamaal, 2018).

Strengthening and deepening primary stakeholders' participation as active participants in interventions is critical for generating accurate, timely, and consistent data, which is imperative for superintending stakeholders' decision-making, encouraging the implementation of effective interventions, and dealing with evolving issues during implementation. This study assessed the utilization of PM&E approaches and how it influences decision making in Maternal and Newborn Health (MNH) programs in Mombasa County.

1.2 Statement of the Problem

Improving maternal, neonatal, and child health (MNCH) is a top goal for global health and human rights. Globally, maternal, neonatal, infant, and under-five mortality rates have decreased over the last three decades; nevertheless, low- and middle-income nations continue to shoulder a disproportionate amount of the burden of morbidity and mortality (Maldonado et al., 2020). Kenya's MMR, NMR, IMR, and under-five mortality rates remain among the highest in the world, at 342 per 100 000 live births, 21 per 100 000 live births, 32 per 1000 live births, and 43 per 1000 live births, respectively (UNICEF, 2020). As of 2017, the facility-based maternal death rate in Mombasa was 195/100,000 live births, the under-five mortality rate was 32.3/1,000, and the infant mortality rate was 57/1,000 (Mombasa County Government, 2018).

Reducing maternal, neonatal, infant, and under-five mortality and strengthening MNCH are key to attaining the SDGs and Kenya Vision 2030. In an environment of high maternal and neonatal mortality, there are both demand and supply side problems. Community stakeholders' capacity to detect maternal and newborn danger indicators, availability to data on maternity and newborn services, nearness to the health facility, and access to transportation are all demand-side obstacles. Supply-side difficulties, on the other hand, include the availability of equipment, supplies, medications, experienced employees, and functioning health systems (Kananura et al., 2017). For example, the 2014-2018 Mombasa County CHSIP final audit identified deficiencies in medical infrastructure and equipment, a lack of competent and motivated personnel, insufficient supply and distribution of medical items, poor quality of medical data, and a lack of health workers' competence and resources to fulfill health system difficulties (Mombasa County Government, 2018).

Participatory M&E systems are critical in detecting these difficulties, which may then be addressed to save lives (Kotch, 2013). Participatory M&E of health programs enables

information gathering and sharing with community stakeholders, healthcare givers, and decision-makers at the county and nationwide levels in order to impact decision-making and action by various stakeholders in order to ensure community emancipation, effective management of health systems, resource allocation based on need, and taking responsibility for meeting health promises (Kananura et al., 2017). However, PM&E is not extensively used in Mombasa County owing to a number of problems, including inadequate funding for the PM&E process, a lack of understanding of the benefits of PM&E, and a negative perception of the entire PM&E process, and a lack of PM&E training for both management and implementers. As a result, the collection of reliable, timely, and consistent data, which is critical for guiding stakeholders' decision-making in MNH initiatives, is hampered.

Kanarura et al. (2017) discovered that PM&E techniques and stakeholder input are highly effective in tracing progress and recognizing developing execution issues, which aids in supporting planning and decision-making throughout implementation. Diverse sources of information and views may aid implementers and decision-makers in understanding evidence and applying it to context for more effective intervention. Kananura et al. (2017) identified the need to improve PM&E skills and resources in order to expand the spaces for decision-making for both key planners and implementers. Another research, Sifunjo (2019), found that PM&E linked to the long-term viability of maternal health programmes.

While there have been studies that have looked at various elements of PM&E in various sectors, a review of prior literature on PM&E of health programs and its role in decision making indicated that there is little information available. Despite the fact that health is a devolved role in Kenya, no known study on PM&E of health programs has been conducted at the county level. None of these researches, in particular, have examined PM&E at the Department of Health, Mombasa County, resulting in a knowledge gap. As a result, the study's aim was to evaluate the use of PM&E methodologies and their effect on decision-

making in MNH in Mombasa County. The study focused on the extent to which Mombasa County Health Department operationalizes PM&E in programs in order to strengthen management and encourage a shift toward a more participatory form of M&E, including documentation of what practices the department is using and, specifically, the use of PM&E in MNH.

1.3 Objectives of the Study

1.3.1 General Objective

Assessment of the utilization of PM&E approaches and its influence on decision making in MNH programs in Mombasa County, with the aim of identifying possible PM&E measures that would be put in place at the initiation, design and implementation phases of MNH programs in order to enhance the quality of decision making and thus enhance the delivery of quality MNH services.

1.3.2 Specific Objectives

1. To establish the influence of utilization of PM&E approaches at the program initiation phase on the quality of decision making in MNH programs in Mombasa County.
2. To assess the influence of utilization of PM&E approaches at the program design and planning phase on the quality of decision making in MNH programs in Mombasa County.
3. To evaluate the influence of utilization of PM&E approaches at the program implementation phase on the quality of decision making in MNH programs in Mombasa County.

1.4 Research Questions

1. To what degree does utilization of PM&E approaches at the program initiation phase influence the quality of decision making in MNH programs in Mombasa County?

2. To what degree does utilization of PM&E approaches at the program design and planning phase influence the quality of decision making in MNH programs in Mombasa County?
3. To what degree does utilization of PM&E approaches at the program implementation phase influence the quality of decision making in MNH programs in Mombasa County?

1.5 Significance of the Study

The Mombasa Health Department's aim is to provide the greatest feasible levels of quality, responsiveness, and comprehensive health care services to all citizens via inventive, efficient, and effective health systems. Community engagement in monitoring and evaluation is critical for achieving meaningful goals. The research shapes a persuasive case to government and non-governmental development actors that active stakeholder engagement in project life-cycles, particularly M&E, is a critical precursor for quality decision making in maternal and newborn health programs.

The findings of this study are valuable to managers and decision makers in the County and various health facilities within Mombasa County in providing critical information for evidence-based policy development to support and sustain PM&E towards enhancing quality decision-making in the provision of MNH services.

Additionally, the study is of great benefit to clients seeking MNH services in public healthcare facilities in Mombasa County as it suggests measures to address identified gaps in PM&E for effective management of health systems, allocation of resources according to need, and ensuring accountability in delivery of health services.

Besides providing a basis for critiquing previous studies in the area of PM&E, this study acts as an empirical source of reference for future studies on PM&E of health programmes and thus build on the knowledge base of PM&E.

1.6 Scope of the Study

This study assessed the utilization of PM&E and its influence on decision making in MNH programs in Mombasa County. The study focused on the utilization of PM&E at the MNH programs initiation, design and planning, and implementation phases. The utilization of PM&E in MNH programs was characterized by consideration of major stakeholders as active partakers in the design and implementation of programs; building the capacity of primary stakeholders to collect data, analyze, reflect, and act; encouraging collaborative learning among stakeholders at various levels; and fueling commitment among stakeholders to taking corrective action(s). The PM&E approaches considered at the MNH programs initiation phase included participatory needs assessment; participatory baseline assessment; and participatory analysis of objectives. The PM&E approaches considered at the MNH programs design and planning phase included participatory feasibility analysis; participatory SWOT analysis; and participatory risk assessment. The PM&E approaches considered at the MNH programs implementation phase included supportive supervision; participatory performance reviews; and participatory desk reviews. Finally, quality of decision making in MNH programs was assessed based on four indicators including decision-making approach, culture, competence and style.

1.7 Limitations of the Study

A descriptive cross-sectional design was used, which limits the study's capacity to make reliable conclusions about the association between the exposure factors and the outcome variable. Because the input and outcome variables were collected concurrently, it may be difficult to distinguish whether the exposure preceded or followed the outcome.

Since cross-sectional studies only study a single moment in time, this study was not able to assess the long-term trends in the utilization of PM&E approaches in MNH in Mombasa County. Additionally, the timeframe selected for this study might possibly not be illustrative of the actual behaviour of the target population as a whole regarding the utilization of PM&E approaches.

The presented observations were derived from self-assessment, self-rating and thus represent perceived effects, and not a randomized trial where health facilities with and without monitoring and evaluation program were compared in terms of objective measures of efficacy and efficiency. This study relies on the associations between the self-reported frequency of using participatory monitoring and evaluation approaches and the frequency of perceived quality decision-making practices in maternal and newborn health programs. We recommend the need for hypothesis driven studies with proper design to strengthen or refute our conclusions.

1.8 Delimitations of the Study

The research was carried out in levels 2 and 3 public health facilities, and community health units in Mombasa County over a period of 6 months. A questionnaire survey involving nurses, community health workers (CHW), maternity patients, facility in-charges, and members of facility management committees was conducted. Additionally, Key Informant Interviews (KIIs) involving county and sub-county reproductive health coordinators were conducted. Only the members of target population meeting the selection standards were included in the sample.

1.9 Assumptions of the Study

The conclusions of the study are predicated on the premise that study participants gave correct data.

1.10 Operational Definition of Key Terms

Decision Making: - Continuous procedures of assessing healthcare conditions or problems, analyzing options, making choices, and carrying out the appropriate measures.

MNH Programs: Programs focusing on health concerns affecting women, children, and families, such as access to appropriate prenatal and antenatal care, newborn screening, infant and maternal mortality prevention, child immunizations, child nutrition, maternal and child mental health, and services for children with special health care needs.

Participatory Monitoring and Evaluation: - The involvement of stakeholders in a program or intervention in initiating, formulating plans, tracking implementation, and assessing performance. This involves the consideration of primary stakeholders as active participants in the initiation, design and planning, and implementation of programs; enhancing the capacity of primary stakeholders in monitoring and evaluation (to collect data, analyze, reflect and take action) through training, joint review meetings, supportive supervision and continuous mentorship; fostering joint information reviews and learning among stakeholders at various phases of the programs; and encouraging stakeholders to be accountable and responsible in taking corrective action(s) based on monitoring and evaluation findings.

Program Initiation Phase: - The initial stage of a program's life cycle, during which an opportunity or need for the project is recognized and a project proposal is produced to address that opportunity or need.

Program Design and Planning Phase: - The second stage of a program's life cycle, in which a plan is developed, identifying the activities, tasks, dependencies, and timeframes, as well as developing a budget that includes labor, equipment, and supply cost estimates.

Program Implementation Phase: - The third stage of a program's life cycle, in which the strategy is executed by organizing and directing project resources to achieve the project's objectives.

Stakeholders: Stakeholders are all parties with an interest in the MNH system and who impact or are affected by the MNH system's policies, decisions, and activities.

CHAPTER TWO: LITERATURE REVIEW

2.1 Introduction

The empirical and theoretical literatures on PM&E are reviewed in this chapter. This chapter also includes a conceptual framework that depicts the connections between the variables under consideration. Finally, the survey highlights knowledge gaps and provides a summary of the literature review.

2.2 Empirical Literature

Sifunjo (2019) examined the impact of PM&E on maternal health programs in Kajiado county, Kajiado North constituency. The study found that participatory project identification resulted in the long-term viability of maternal health programmes. The study also found that stakeholder engagement in vision, mission, and target setting improved the success of maternal health programs. The study also discovered that identifying and involving stakeholders has a beneficial impact on the performance of maternal health programs.

Mohammed, Alhassan, and Kanlisi (2018) assessed stakeholder engagement in district assembly project and program monitoring and evaluation in Ghana. The authors established that stakeholder engagement in project and program M&E was high at the Municipal and District levels, but low at the zonal, council and community levels. This had adverse influence on openness, accountability, and the long-term viability of initiatives and programs.

Jamaal (2018) investigated the influence of PM&E on project performance at the Kenya Marine and Fisheries Research Institute. According to the author, PM&E processes engage stakeholders in collaborative planning and progress evaluation, which results in project success; the feasibility of a project is typically tied to financial capital; participatory M&E practices community financial mobilization, which leads to project success; and total quality management projects require rigorous pre-planning, which leads to project success.

Kananura et al. (2017) investigated how participatory M&E techniques aided in the identification of critical design and implementation difficulties, as well as how they affected decision-making among stakeholders in eastern Uganda. Data were gathered through a reflective examination of several M&E procedures employed in a MNH initiative executed in three areas in eastern Uganda. The results showed that the PM&E techniques were beneficial at the design stage for detecting major local challenges and potential home-grown solutions, as well as shaping the actions that were later executed. At the implementation phase, the PM&E approaches provided factual data that formed the basis for decision-making and assisted in the identification of evolving issues, for instance, poor execution by certain village health teams, constrictions at the health facility such as poor usage of standardized guidelines, a lack of pits at certain facilities for placenta disposal, insufficient gasoline for the ambulance, and lack of proper care for infants with low birth weight. When these facts were shared with essential stakeholders, it spurred them to take necessary action. The leadership at the sub-county, for instance, built pits for placenta disposal, the district health officer supplied gasoline for ambulances, and health providers got refresher training and mentorship in infant care.

Otieno, Munyua, and Olubandwa (2016) evaluated the influence of PM&E on the implementation of the LASDAP project in Bondo Sub-County. According to the findings, PM&E in general enriched the LASDAP process, particularly relationships among stakeholders and the quality of project output. PM&E also improved good governance by increasing accountability, sensitivity to people' demands, and openness.

Kajaga (2016) investigated how much PM&E improves service delivery in Uganda. The research was conducted in the Gulu District as part of the USAID-SAFE initiative. The study discovered a considerable impact of participatory planning, execution, and decision making on service delivery at the USAID-SAFE program in Gulu. The study indicated that including

stakeholders in M&E operations for a project improves service delivery and project sustainability.

Karanja (2016) investigated the use of PM&E in the management of CDF projects in Dagoretti South Sub-County. The findings demonstrated that stakeholders were not sufficiently involved in CDF project M&E, and their engagement was very minimal at all phases of the PM&E process. The documentation of actions linked to stakeholders' engagement in project M&E was weak. Furthermore, project management committees' M&E capability and community knowledge of CDF project monitoring and evaluation were lacking.

2.3 Research Gaps

While there have been studies that have looked at various elements of PM&E in various sectors, a review of prior literature on PM&E of health programs and its role in decision making indicated that there is little information available. The following table summarizes the research gaps and the contributions of the current study.

Table 2.1: Research Gaps

Author / Year	Research Title	Findings	Research Gaps	Contributions of the Current Study
Sifunjo (2019)	Participatory M&E and successful implementation of maternal health projects within Kajiado north constituency, kajiado county	Participatory project identification led to sustainability of maternal health projects. Stakeholder's participation in vision, mission, and objectives setting positively affected the performance of the maternal health projects. Stakeholder identification and involvement positively affected the performance of the maternal health projects.	Did not focus on the influence of PM&E approaches on decision making.	This study assessed the effect of PM&E approaches at the initiation, design & planning, and Implementation phases on decision making in MNH programs
Mohammed, et al.	Stakeholder engagement in	Stakeholder engagement in project and program M&E was high among	Did not focus on	This study assessed the

(2018)	M&E of district assembly projects and programs in Ghana.	municipal & district levels, but low at zonal & community levels. This had a detrimental influence on openness, accountability, and the long-term viability of initiatives and programs.	the influence of PM&E approaches on decision making.	effect of PM&E approaches at the initiation, design & planning, and Implementation phases on decision making in MNH programs
Jamaal (2018)	Effects of PM&E on project performance at KEMFRI.	The PM&E process engages stakeholders in collaborative planning and progress analysis, which results in successful project completion; financial capital is frequently tied to project viability. PM&E delivers financial mobilization methods by communities that lead to project success, and overall quality management projects necessitate rigorous pre-planning that leads to project performance success and impact change in its everyday practice.	Did not focus on the influence of PM&E approaches on decision making.	This study assessed the effect of PM&E approaches at the initiation, design & planning, and Implementation phases on decision making in MNH programs
Kananura et al. (2017)	PM&E approaches that influence decision-making: lessons from a maternal and newborn study in Eastern Uganda	The PM&E methodologies were effective at the design stage for detecting major local challenges and potential local solutions, as well as shaping the undertakings that were later executed. At the implementation phase, the PM&E methodologies provided facts that formed the basis for decision-making and assisted in the identification of evolving issues, for instance, poor execution by certain village health teams, constraints at the health facility such as inadequate utilization of standard guidelines, limited pits at certain facilities for placenta disposal, deficient gasoline for the ambulance, and lack of proper care of infants with low birth weight infants. When this facts were shared with critical stakeholders, it drove	The study was not conducted in Mombasa County	The study was carried out in Mombasa County with the aim of identifying possible PM&E measures that would be put in place at the initiation, design and implementation phases of MNH programs in order to improve decision making and thus enhance the delivery of quality MNH services.

		them to undertake essential action.		
Otieno et al. (2016)	Effect of participatory M&E on the LASDAP project implementation in Bondo sub-county.	In general, PM&E enhanced the LASDAP process, particularly stakeholder relationships and project output quality. PM&E also improved good governance by increasing accountability, responsiveness to people' demands, and openness.	The study was not conducted in health sector.	The current study was conducted in public health facilities and community units in Mombasa County with a focus on MNH programs.
Kajaga (2016)	PM&E and service delivery in Uganda: A case of USAID-SAFE program in Gulu district	The study discovered that PM&E and participative decision making had a substantial impact on service delivery at the USAID-SAFE program in Gulu.	Did not focus on the influence of PM&E approaches on decision making.	This study assessed the effect of PM&E approaches at the initiation, design & planning, and Implementation phases on decision making in MNH programs
Karanja (2016)	Evaluation of the PM&E approach: A case study of CDF projects in Nairobi's Dagoretti South Sub-county.	Stakeholders were not sufficiently included in the M&E of CDF projects, and their engagement in all levels of the PM&E process was very low. The documentation of actions relating to stakeholder engagement in project M&E was deficient. Furthermore, project management committees' M&E capability and community understanding of CDF project monitoring and evaluation were lacking.	Did not focus on the influence of PM&E approaches on decision making.	This study assessed the effect of PM&E approaches at the initiation, design & planning, and Implementation phases on decision making in MNH programs

2.4 Participatory Monitoring and Evaluation

Hilhorst and Guijt (2006) observed that PM&E emphasizes tracking and feedback (monitoring), valuing and performance appraisal (evaluation), and expanding and developing the involvement of stakeholders (participation). As a developing discipline, PM&E has been progressively implemented in many settings and hundreds of development programs

throughout the world, gaining diverse roles as individuals and organizations acquire more knowledge on how to acclimatize, transform, and experiment with different participatory methods (Onyango, 2018). The PM&E process is therefore focused on identifying how to evaluate progress, agreeing on expected outcomes and milestones, acquiring appropriate data, performing collaborative analysis, and deciding on actions (Hilhorst & Guijt, 2006). As a result, the primary phases in putting PM&E into reality are: creating a PM&E process; data collection and analysis; and documenting, reporting, and information sharing.

2.4.1 Planning for a PM&E Process

Since it necessitates a protracted process of negotiation, debate, and joint decision making among multiple stakeholders, the planning stage is often regarded as the most fundamental to the success and efficacy of the PM&E process. At this point, several stakeholder groups gather to express their problems, needs, and expectations (Luutu, 2016).

At the outset, it is necessary to identify and choose the stakeholder groups that will be participating in the PM&E process planning. These are those who have an impact on or are impacted by a development program and have fundamental rights as citizens to voice their views on public problems. It is critical to construct structures that enable engagement with interested parties and the creation of a diverse variety of participatory procedures (Mohammed et al., 2018). An analysis of stakeholders is performed to discover their interests and responsibilities in the development process. There is significant benefit in including stakeholders in order to increase ownership of the development endeavor. Mulwa (2008) states that while determining who should participate, questions to ask include how participants will be identified and selected, their backgrounds and interests, and who should and wants to be a part of the process. Changes in stakeholders' behaviors, attitudes, knowledge, and skills that are required in the PM&E process will also be taken into account.

At this point, defining the PM&E project's objectives is crucial. This approach should include extensive talks amongst stakeholders about the various parties' requirements and aims (Luutu, 2016). To identify the objectives of PM&E, one must first understand who the participants in the process are, as well as who the end-users will be, why the project is being carried out, and how the outcomes and process will be used. Community members, project (local) personnel, donors, development agencies, research, national level policymakers (GOs and NGOs), and even indirect beneficiaries in a community or the broader public are all examples of end-users. What is to be monitored and evaluated, as well as how the process is to be used, will be determined primarily by who need PM&E findings and information, as well as who is engaged in creating the process (Onyango, 2018).

After agreeing on PM&E objectives, indicators may be created. In general, indicators are distinct from objectives in that indicators are considered to be relatively concrete and particular, but objectives may represent the project's or initiative's larger overall viewpoints, representing its values, ambitions, and long-term aims (Bell & Aggleton, 2016). The choice of indicators will ultimately be determined by what is being assessed, who the end users are, and how the information will be used. Many people believe that a consultation process among many stakeholders to define objectives and indicators is vital to properly carrying out and maintaining PM&E (Glandon et al., 2017).

2.4.2 Collection and Analysis of PM&E Data

Following the identification of information needs and objectives, the next important phase in the PM&E process is selecting how to collect and gather information. A wide range of methods and approaches can be employed to collect data. The selection of tools will be heavily influenced by the environment and project-specific requirements. Several critical issues must be addressed at this point, including where the information will be collected,

which technologies should be utilized, and who will acquire the information and when (Bell & Aggleton, 2016).

According to Glandon et al. (2017), actively incorporating stakeholders in data collecting helps foster ownership of the development project while also producing more credible monitoring outcomes. Stakeholders can utilize a variety of participatory approaches, including visual (observation), interviews, group instruments and exercises, such as mapping, ranking, community scorecards, and PRA tools and methods, such as Community animators (Mulwa, 2008).

Following data collection, the subsequent step of PM&E comprises processing or analyzing gathered data, while ideally, analysis should occur throughout the PM&E cycle (Bell & Aggleton, 2016). Defining the scope of data analysis, particularly what is to be studied, how, and by whom, is an important part of creating PM&E. (Rossman, 2015). The methods or procedures used to collect data, as well as the information requested by participants, will determine how data is analyzed (Glandon et al., 2017).

While analysis of data is usually considered as a task that is mechanical and driven by experts, PM&E should be regarded as a chance to actively engage different categories of project stakeholders in the critical appraisal of achievements and limitations, as well as the drawing of inferences and lessons learned (Onyango, 2018). The goal of PM&E is to incorporate all consumers and stakeholders – as well as beneficiaries – in analyzing data at all levels. At this level of PM&E, stakeholders take part in serious consideration and thought regarding the challenges and limits, as well as the accomplishments and consequences of their efforts and activities. Some of the topics covered in information analysis, according to Scott- Glandon et al. (2017), are; analysis of the significance of program objectives in relation to the needs of

stakeholders; reviewing the influence of the activities carried out; and assessing the methodology through which the program was conducted and how decisions were arrived at.

2.4.3 Dissemination of PM&E Results

In contrast to conventional M&E, the final step of PM&E comprises documenting, reporting, and sharing of findings created by the PM&E process, as well as relating them to action. Participatory M&E is fundamentally about information exchange amongst program recipients, program executors, donors, and, in certain instances, external evaluation experts (Rossman, 2015).

Records of acquired data are presumably retained from the beginning of the project in order to give a 'holistic' picture during the course of the endeavor. Regardless of how participatory the M&E process has been in the preceding processes, not all stakeholders can be included in M&E data gathering and analysis. The outcomes of M&E operations are shared with other stakeholders in this stage, and appropriate actions are deliberated based on the outcomes (Onyango, 2018).

At this point, numerous critical concerns must be addressed, particularly those concerning the ownership and use of information and discoveries. According to Glandon et al. (2017), clear guidelines should be developed for how information will be utilized and communicated, particularly when a number of parties with varying demands and interests are involved. Most information, for example, is distributed in written form or in a more formal style, which may not be suited for reaching local audiences. As a result, a strong focus is made on conveying PM&E outcomes and conclusions in a variety of methods that react to end-user demands. Forms of communication may include more casual reporting forms, such as speaking the local language or employing more graphic tactics (Mulwa, 2008).

2.5 Participatory M&E and Decision Making in MNH

Participatory M&E involves main stakeholders in decision making by allowing them to drive the M&E process, improve their abilities, and, when necessary, support them in making their own decisions on problems that impact them (Kananura et al., 2017). This might have a variety of advantages. It, for example, contributes to the future development of local capabilities and improves people's capacity to monitor and control their own growth. This, in turn, promotes their ownership of initiatives and programs. Furthermore, it aids in ensuring that any advantages accrued once a project or program is completed, are retained within the community of interest. The health-care system has grown increasingly conscious of the significance of including communities and users in the administration of health-care institutions and the evaluation of service delivery. One option is the formation of 'health facility management committees,' which are charged with garnering local support, mobilizing funding for operating expenditures and upkeep, and establishing relationships with communities and end-users (WHO, OECD & World Bank, 2018; Waweru et al., 2013).

In Kenya, for example, level 2 and 3 public health institutions are supervised by Facility Management Committees. Each public primary health care facility has a health facility management committee comprised of nine (9) elected members. These committees' membership includes the facility in-charge (who serves as the committee's secretary and is ex-officio), an area Member of County Assembly, a ward administrator, a religious leader, a youth representative, a community representative, a business representative, a women representative, and a disability representative. The committee meets regularly and is in charge of mobilizing resources, approving facility budgets and procurement strategies, and ensuring that community requirements are addressed. Furthermore, these committees must actively monitor and evaluate the effectiveness, efficiency, and equality of service delivery.

Participatory M&E ensures that resources are distributed in accordance with the requirements of individuals or populations. Need-based resource allocation is the practice of allocating resources to people or communities based on their access to health-care services. Equitable allocation of resources is regarded as being among the primary challenges of any system of health in any region of the world, and it is also recognized as a critical component of decision-making in this respect (Ransom & Olsson, 2017). As a moral problem, it plays essential roles in establishing fairness in health care services and in the health repercussions of those treatments. When health care resources are divided fairly across competing customers (e.g., regions) based on their health-care demands, resources are allocated fairly (Daniels, 2016). As a result, need-based allocation of resources is among the techniques that have been examined for fair resource allocation in most publicly supported health care systems in recent decades (Ransom & Olsson, 2017). Using this strategy, it is possible to ensure that the government's public resources are dispersed fairly to various areas in accordance with the health system's aims.

Participatory M&E results in context-specific health treatments. A contextualized intervention is one that has been customized to the local context by gathering data from targeted recipients and using this data to tailor the intervention to the unique requirements of the population (Daniels, 2016). Beneficiaries of interventions are frequently in the greatest position to raise pertinent questions, recognize changes in their local communities and surroundings, and understand how initiatives or programs are affecting them (Kananura, 2017). They are also the finest people to know if and how initiatives or programs may be enhanced. Involving them in PM&E allows for a more accurate view of the situation on the ground. Because PM&E is generally continuous, timely, dependable, and appropriate information may be fed back into decision-making, allowing a project or program to quickly adjust and improve (Glandon et al., 2017). Participatory M&E may also improve the targeting of development interventions by

making sure that activities are grounded on home-grown knowledge and awareness of problems, and so are applicable to local needs (Kananura et al., 2017).

Participatory M&E can be utilized to influence policy development choices. Findings-informed health policymaking is a policymaking strategy that seeks to ensure that policy decisions are influenced by the greatest available research evidence (Lubita et al., 2017). How this is done will vary depending on the sort of choice being made and the situation. Nonetheless, evidence-informed policymaking is distinguished by systematic and interactive access to and evaluation of evidence as an input into the policymaking process. This does not mean that the policymaking process as a whole will be methodical and transparent. However, methodical techniques are utilized within the entire policymaking process to guarantee that relevant information is located, assessed, and used effectively (Glandon et al., 2017). These processes are open to the public, allowing people to assess what research evidence has been utilized to drive policy choices, as well as the judgments made about the evidence and its consequences. Policymakers must have access to credible evidence in order to make well-informed choices regarding topics such as how to offer universal and equitable access to healthcare. Evidence is required to establish what services and programs will be offered or covered, how those services will be delivered, financing arrangements, governance arrangements, and how change will be implemented (Onyango, 2018).

To aid in the making of sound decisions, Bujar et al. (2016) and Donelan et al. (2015) created ten Quality Decision-Making Practices (QDMPs). These were developed based on the outcomes of semi-structured interviews with 29 key opinion leaders from regulatory agencies and pharmaceutical businesses in order to examine and identify the critical aspects influencing quality decision making (Donelan et al., 2015), and were thought to be pertinent to those two stakeholders (Bujar et al., 2016). Structure and Approach, Evaluation, Impact,

and Transparency and Communication are the four areas in which the ten QDMPs are arranged (Bujar et al., 2016).

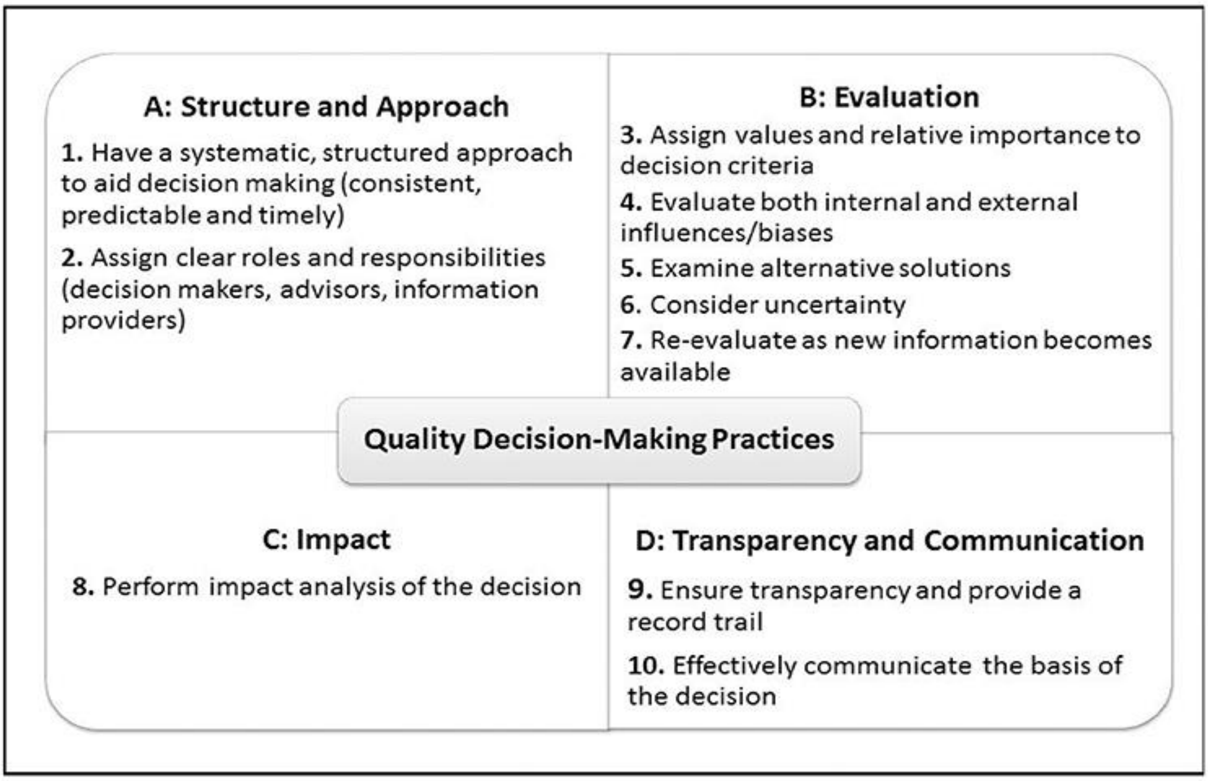

Figure 2.1: The 10 QDMPs

2.6 Theoretical Framework

2.6.1 Programme Theory

A program theory is a set of explicit and implicit assumptions held by stakeholders about how change will occur and how an intervention will achieve these causal processes. It describes how an intervention is assumed to give rise to a series of outcomes that result in the desired or actual impacts. It might encompass both positive (useful) and negative (harmful) effects (which are detrimental). It can also highlight other aspects that contribute to the production of affects, such as context and other initiatives and programs (Funnell & Rodgers, 2011).

A theoretical framework for monitoring, evaluation, or an integrated M&E framework can be provided by program theory. A program theory could be a very valuable means of consolidating existing knowledge regarding a program and identifying points of consensus and differences about the way the program is thought to function, as well as where evidence lacunas exist. It could be utilized for a single assessment, arranging group evaluations of a number of projects sponsored by the same program, or bringing together findings from numerous assessments (Funnell & Rodgers, 2011).

A PM&E approach based on program theory would determine how an intervention is thought to operate and what intermediate outcomes must be met for the intervention to be effective. This enables PM&E stakeholders to discern between implementation failure (not done correctly) and theoretical failure (done right but still did not work). It is hard to tell if the PM&E stakeholders have assessed the correct components of implementation quality and quantity without program theory (Funnell & Rodgers, 2011).

2.6.2 Empowerment Evaluation Theory

Empowerment evaluation is an assessment approach that aims to increase the likelihood of program goals being met by (1) providing assessment tools to program stakeholders for assessing program planning, execution, and self-evaluation, and (2) ensuring that evaluation is incorporated into program/organization planning and management (Fetterman, 1994). It is a process that involves stakeholders and strives to give communities with the tools and information they need to monitor and analyze their own performance and accomplish their goals. It focuses on real-world problem solving as well as programming enhancements and consequences. Empowerment evaluation focuses on fostering self-determination and sustainability, and it is especially well suited to assessing big community-based or place-based projects (Fetterman, 2015).

2.6.3 Theory-Driven Evaluation Theory

Theory-driven evaluation is a contextual or comprehensive examination of a program based on program theory's conceptual framework. It was created in response to policy and program assessment techniques that were either confined to before-and-after and input-output designs or focused solely on methodological difficulties (method-driven evaluation). The goal of theory-driven assessment is to examine not only the efficacy of an intervention (whether it works or not), but also its causal mechanisms (how and why it works), while keeping the intervention's context in mind. The data is critical for parties looking to enhance current or future initiatives (Chen, 2012).

The program theory is the essential component of this method. A program theory can be described for any intervention. This hypothesis describes how the intervention's planners anticipate it will achieve its goal. The program theory is a hypothesis that may be evaluated and developed further (Chen, 1990). Program theory, as a foundation for building theory-driven assessment, is a systematic organization of stakeholders' explicit and tacit prescriptive and descriptive assumptions that drive programs (Chen, 2005). Descriptive assumptions, often known as change models, address what causal processes are expected to occur in order to achieve program objectives. Prescriptive assumptions, often known as action models, address what activities must be conducted in a program in order to create anticipated results. The action model and change model are used in theory-driven assessment to address contextual elements as well as planning and implementation concerns that are critical to program effectiveness (Chen, 2012).

2.7 Conceptual Framework

The study conceptualized that utilization of PM&E approaches in MNH programs initiation, design and planning, and implementation phases, would lead to improved decision making among stakeholders in MNH programs. The PM&E approaches considered at the MNH programs initiation phase included participatory needs assessment; participatory baseline assessment; and participatory analysis of objectives. The PM&E approaches considered at the MNH programs design and planning phase included participatory feasibility analysis; participatory SWOT analysis; and participatory risk assessment. The PM&E approaches considered at the MNH programs implementation phase included supportive supervision; participatory performance reviews; and participatory desk reviews. Finally, the quality of decision making in MNH programs was assessed using modified Quality Decision Orientation Scheme (QoDoS), which is a standardized tool for measuring the quality of decision making (Donelan et al., 2016; Bujar et al., 2017). The modified QoDoS was used to assess quality of decision making at both the organizational level and individual level using four indicators. Two of the indicators, decision-making approach and decision-making culture, measured quality of decision making at the organizational level, while decision-making competence and decision-making style measured quality of decision making at the individual level. The modified QoDoS is an effective tool of assessing Quality Decision Making Practices (QDMPs) because it assesses the practices of both individuals and organizations.

Table 2.2 shows how the variables were operationalized.

Table 2.2: Operationalization of Variables

Variable	Type	Indicators	Scale
Utilization of PM&E approaches at the MNH programs initiation phase	Independent	▪ Participatory needs assessment.<br>▪ Participatory baseline assessment.<br>▪ Participatory analysis of objectives.	5-point Likert scale
Utilization of PM&E approaches at the MNH programs design and planning phase	Independent	▪ Participatory feasibility analysis<br>▪ Participatory SWOT analysis<br>▪ Participatory risk assessment	5-point Likert scale
Utilization of PM&E approaches at the MNH programs implementation phase	Independent	▪ Supportive supervision<br>▪ Participatory performance reviews<br>▪ Participatory desk reviews	5-point Likert scale
Quality of decision making in MNH programs	Dependent	▪ Decision-making approach<br>▪ Decision-making culture<br>▪ Decision-making competence<br>▪ Decision-making style	5-point Likert scale

Nayak and Singh (2015) define a conceptual framework as a illustrative depiction showing the connection between study variables. In this study the independent variables are, PM&E approaches in MNH programs initiation phase, PM&E approaches in MNH programs design and planning phase, and PM&E approaches in MNH programs implementation phase. The dependent variable is decision making in MNH programs as shown in Figure 2.1.

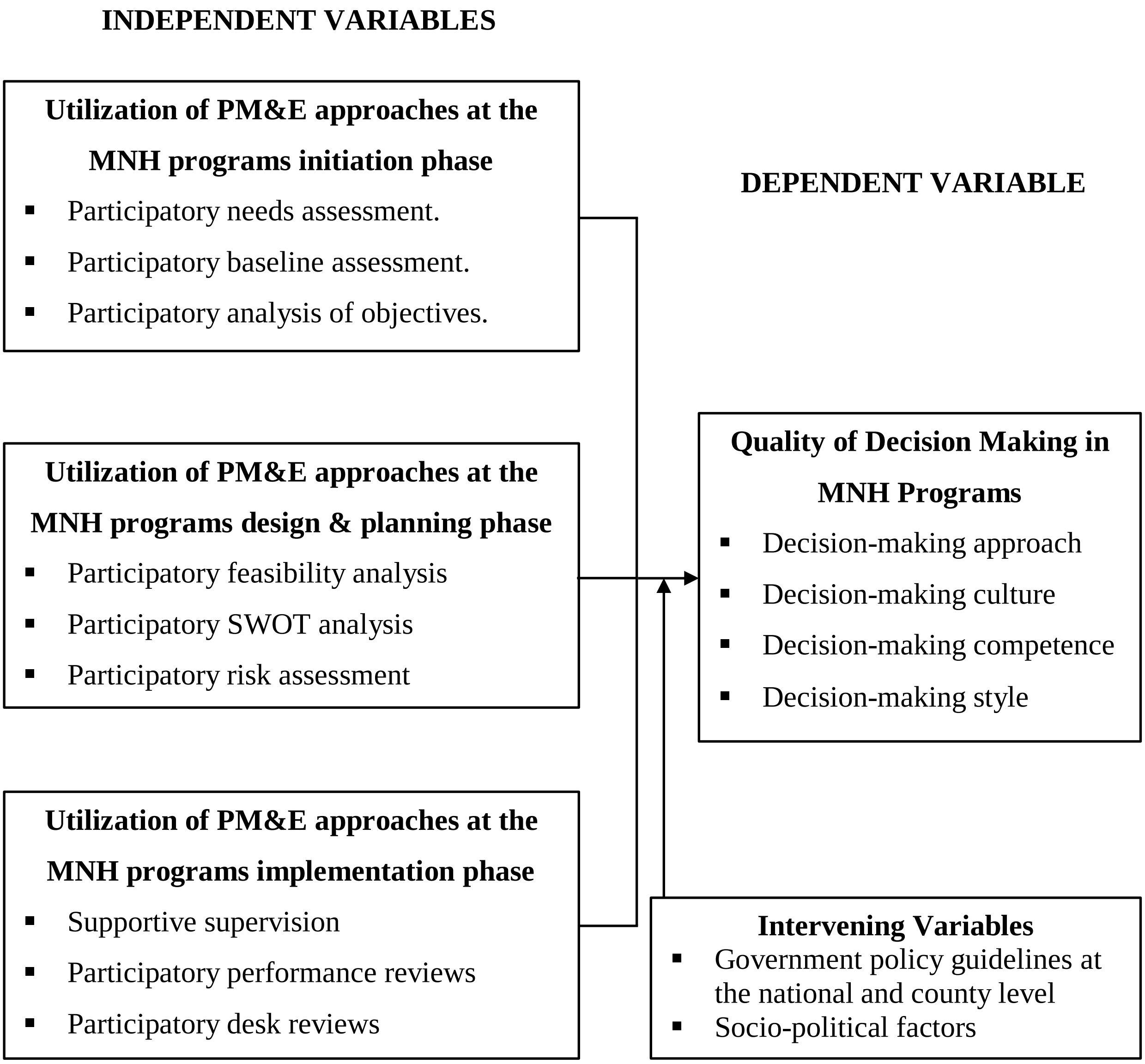

Figure 2.2: Conceptual Framework

2.8 Recap of Literature Review

Monitoring, evaluation, and enhancing and entrenching stakeholder engagement are all aspects of participatory M&E. Planning for a PM&E process; data collection and analysis; and documenting, reporting, and sharing of PM&E results, as well as necessary action, are the primary phases in putting PM&E into effect. Previous research has shown that the PM&E methodologies are beneficial for identifying major local challenges and potential local solutions at the program initiation phase. The PM&E methodologies are used to inform the

actions that are later conducted throughout the program design and planning phases. At the program implementation phase, the PM&E methodologies give factual data that provides the basis for decision-making and assists in the identification of emergent difficulties. When the factual data is shared with essential stakeholders, they are encouraged to implement evidence-based solutions.

CHAPTER THREE: RESEARCH METHODOLOGY

3.1 Introduction

The processes of the study are explained in this chapter, which includes the research methodology, research design, population and sampling, data collection, reliability and validity, and data analysis methodologies.

3.2 Research Methodology

A mixed methods research technique was used. Mixed methods research is a research methodology in which the researcher blends parts of qualitative and quantitative research methodologies to get a broader and deeper knowledge of phenomena and to corroborate results (Johnson & Christensen, 2017). That is, the researcher gathers and analyzes both quantitative and qualitative data in order to answer the study's research questions. The research used a QUAN + qual mixed method approach to analyze the use of PM&E approaches in MNH programs in Mombasa County, as well as assess the effect of PM&E approaches on the quality of decision making. This triangulation of research methodologies looks for convergence, corroboration, and correlation of results from several approaches.

3.3 Research Design

A descriptive cross-sectional design was used looking at the use of PM&E methodologies and their impact on decision making in MNH programs in Mombasa County, utilizing information gathered from key stakeholders at level 2 and 3 health institutions and community health units. This design was used since it is less time-consuming and hence meets the time constraints of this investigation. The design also enables a researcher to collect data from a large number of patients and compare variations across groups (Kothari & Gaurav, 2014).

3.4 Location of the Study

Mombasa County is an island in Kenya's coastal area that has 65 square kilometers of Indian Ocean water. It is bounded on the north by Kilifi County, on the south by Kwale County, and on the east by the Indian Ocean. According to the 2019 population census, the county has a population of 1,208,333 people, of which 610,257 are male and 598046 are female, and is the smallest in the country, occupying an area of 229.7 square kilometers and. Changamwe/Jomvu, Kisauni/Nyali, Likoni, and Mvita are the county's four sub-counties. Because it is an urban city county, it has a huge population of both local and immigrant groups who come in search of job, education, and investment possibilities. This means that health infrastructure and services, especially MNH, will be under increased strain. Improving MNH is one of the most difficult tasks in the county's health-care delivery. As of 2017, the county's facility-based maternal mortality rate was 195/100,000 live births, the under-five death rate was 32.3/1,000, and the infant mortality rate was 57/1,000. (Mombasa County Government, 2018).

Based on the scale and complexity of the services provided, the County's health system is organized around six levels of care that fall into four tiers of care. The first layer (Level 1) is structured in community health units of around 100 families or 5,000 community members, which are managed by CHWs, volunteers who are overseen by community health extension workers (CHEWs), who are employed by the Ministry of Health (MOH). The second and third tiers (Levels 2 and 3) are made up of primary care health facilities with dispensaries and health centers staffed by nurses and clinical officers. The county referral facilities, which comprise the previous primary and secondary hospitals, make up the third tier (levels 4 and 5). The fourth tier (level 6), the national referral institution, provides highly specialized care as well as training and research assistance. Government-owned/public facilities, faith-based groups, and private health institutions are among the health facilities in the many levels of

care. The community health units and public primary health care facilities categorized into levels 2 and 3 were the subject of this investigation.

3.5 Target Population

The primary target population included 1500 CHWs in community health units; and 120 nurses, 570 maternity patients (6 months population estimate based on the number of deliveries between July to December 2021), 36 health facility in-charges and 288 health facility management committee members in 36 levels 2 and 3 public health facilities (Mombasa County, 2020). Data collected from this population formed the primary basis for analysis. Health facility in-charges, nurses and CHWs were chosen as the primary target population for this study because they are involved in the daily operations of the health facilities/community health units and therefore poses first-hand experience and knowledge on PM&E process at the public health facilities/community health units. They are also the primary producers and consumers of the PM&E products (they are on both sides of the demand and supply). Maternity patients were involved because they are the end-users of the services and therefore are an important stakeholder group in PM&E. Levels 2 and 3 public health facilities are managed through Facility Management Committees, whose members are drawn from the community and include area Member of County Assembly, ward administrator, religious leader, youth representative, community representative, business representative, women representative, disability representative, and facility in-charge (who is the secretary to the committee and ex-officio). The committee members are responsible for resource mobilization, approving facility budget and procurement plans, and ensuring community needs are met. They are therefore conversant with the PM&E processes in the facilities.

The secondary target population comprised of 7 county and sub-county reproductive health coordinators drawn from the six sub-counties in Mombasa County (Mombasa County, 2020).

The reproductive health coordinators are involved in the coordination and implementation of policies, regulations and obligations relating to MNH service delivery. Therefore, they are well conversant with the PM&E processes in the county. They were involved in this study as key informants in providing in-depth information on PM&E approaches utilized in the county department of health.

Table 3.1: Target Population

**Category**	**Population Size**
Nurses	120
CHWs	1500
Maternity Patients (6 months estimate)	570
Health Facility Management Committee Members	288
Health Facility In-charges	36
County and Sub-County Reproductive Health Coordinators	7
**Total**	**2521**

3.5.1 Inclusion Criteria

i. Nurses, health facility management committee members, and in-charges in level 2 or 3 public health facilities, and CHWs in community health units in Mombasa County.

ii. Patients aged between 15-49 years who sought maternity health services in level 2 and 3 public health facilities in Mombasa County during the period of the study.

iii. County and sub-county reproductive health coordinators.

iv. Those who voluntarily granted their consent to be study participatants.

3.5.2 Exclusion Criteria

i. Nurses, CHWs and facility management committee members who had worked in the study facility/unit, for less than six months prior to the study.

ii. Nurses from the selected health facilities who were absent from duty during data collection period.

iii. Nurses, CHWs, facility management committee members, maternity patients, and reproductive health coordinators who did not give consent.

3.6 Sampling Procedures and Techniques

All the county and sub-county reproductive health coordinators were selected for the study as key informants. Additionally, health facility in-charges from all the levels 2 and 3 public health facilities were included in the sample of the study.

The stratified random sampling approach was used to pick nurses, CHWs, maternity patients, and members of the health facility management committee. This sampling approach was used in the study to guarantee that the four categories are appropriately represented in the study's sample (Kothari and Gaurav, 2014). As a result, the four groups (nurses, CHWs, maternity patients, and members of the health facility management committee) comprised the study's stratum. The study then used a proportionate allocation approach to distribute the sample size among the various strata in accordance to their sizes (Sample Size/Population Size) x Sub-group Size).

After apportioning the sample size to each stratum, the study adopted systematic random sampling in order to provide the members in each stratum an equal opportunity to participate in the study (Kothari & Gaurav, 2014). The population size per stratum was divided by the corresponding stratum sample size to obtain the sampling interval (nth). For nurses, CHWs and health facility management committee members' strata, the first participant was selected by randomly picking folded papers containing identity numbers of the stratum members as per a list that was obtained through the department of health, Mombasa County. Thereafter, every nth member, as per the list, who met the inclusion criteria was selected. This process was

done separately for each stratum to obtain the required stratum size. For maternity patients, the first patient to seek maternity services in each of the selected health facilities, during the period of study, constituted the first participants. Thereafter, every nth patient, meeting the selection criteria, was included in the sample. The numbers of selected maternity patients from the various facilities were summed up each day and the process was completed once the stratum sample size was attained.

3.7 Sample Size

All the 7 county and sub-county reproductive health coordinators were included in the study as key informants.

Yamane's (1967) formula was used to calculate the sample size comprising of in-charges, nurses, CHWs, maternity patients, and health facility management committee members.

$$n = \frac{N}{1 + N(e)^2}$$

Where n = Sample size;

N = Population size; and

e = Significance level

Therefore, the calculated sample size for nurses, CHWs, maternity patients and committee members was;

$$n = \frac{2514}{1 + 2514(0.05)^2} \cong 345$$

To account for a 10% potential withdrawal or non-response rate, the predicted sample size was increased. The calculated sample size, n, was divided by (1-w), where w was the projected percentage of withdrawal or non-response.

$$n^{**} = \frac{n}{1-w}$$

Therefore, the adjusted sample size was;

$$n^{**} = \frac{345}{1-0.1} = 383$$

The 36 health facility in-charges from all the level 2 and 3 public health facilities were included among the 383 study participants. The distribution of the remaining 347 participants among nurses, CHWs, maternity patients and health facility management committee members was determined using proportionate sampling, based on the population in each group.

$$\text{Proportionate sample size} = \frac{\text{Sample size}}{\text{Population size}} \times \text{Sub} - \text{group size}$$

Table 3.2: Distribution of the Sample

**Category**	**Population Size**	**Sample Size**
Nurses	120	17
CHWs	1500	210
Maternity Patients	570	80
Health Facility Management Committee Members (Excluding in-charges)	288	40
Health Facility In-charges	36	36
**Sub-Total**	**2514**	**383**
County and sub-county reproductive health coordinators	7	7
**Total**	**2521**	**390**

3.8 Construction of Research Instruments

A questionnaire survey using structured-questionnaire (Appendix 2) was conducted to collect primary data. The questionnaire was used to collect data on the socio-demographic information and the three independent variables including PM&E approaches at the MNH programs initiation phase, PM&E approaches at the MNH programs design and planning phase, PM&E approaches at the MNH programs implementation phase, and quality of

decision making. The contents of the questionnaire on the independent variables were informed by a thorough literature review and the expert guidance of the supervisors. The questionnaire was fine-tuned based on feedback from the pre-test findings and expert input of the supervisors. Additionally, the study adopted a modified QoDoS (Donelan et al., 2016; Bujar et al., 2017) to collect data on quality of decision making in MNH programs. The modified QoDoS assessed four indicators of quality decision making including decision-making approach, culture, competence and style. The modified QoDoS is an effective tool of assessing QDMPs because it assesses the practices of both individuals and organizations.

Key Informant Interviews (KIIs) using interview guides (Appendix 2) were conducted to collect qualitative data. The interview guides comprised key questions that informed comprehensive, systematic and in-depth discussions on PM&E and its influence on decision making. This tool collected information on involvement of stakeholders in PM&E processes, adoption of PM&E approaches at the design, planning and implementation phases of MNH programs at the county, impact of PM&E adoption on decision making in MNH programs, and suggestions for improvement of PM&E process at the facility level and the department of health at the county level.

3.9 Pre-testing, Validity and Reliability

3.9.1 Pre-testing

Pre-testing of the research instruments and process was conducted in 4 levels 2 and 3 public health facilities in Kilifi County (10% of the actual study facilities). This assisted in assessing and testing adequacy of instruments used in the study as well as feasibility of the study itself. The sample for the pre-test comprised 39 respondents (10% of the actual sample size) as recommended by Nayak & Singh (2015), including 1 reproductive health coordinator, 4 facility in-charges, and 34 nurses, CHWs, facility management committee members and maternity patients. Once the pre-test is done, data were reviewed and analyzed. In case of

suggestions and comments by the respondents, they were looked at and improvement made. Comprehension, suitability, and sequencing of the questions were done as well as assessing the time taken to complete each questionnaire, and appropriate adjustments were made and the tools refined.

3.9.2 Validity

The capacity of study findings to properly represent what the research sought to assess so that inferences may be drawn to the full population is referred to as validity (Kothari & Gaurav, 2014). To guarantee that the findings of a measure accurately mirror those of the desired variable, data collecting instruments were enhanced for precision, clarity, and inclusivity. Validity was verified by verifying that the questionnaires adequately covered the issues under research and that the instruments contained a representative sample that could be extrapolated to the rest of the population. Criterion validity was assessed by ensuring that the information requested by the criterion is gathered. The face validity of the surveys was checked to ensure that they measure the characteristic of interest. The formulation of questions that were not scored in the YES or NO direction, as well as the usage of questions that did not generate defensiveness, also boosted validity.

3.9.3 Reliability

An instrument’s ability to regularly generate identical outcomes is referred to as its reliability (Kothari and Gaurav, 2014). Cronbach's alpha (1951) was used in the study to assess the internal consistency of a group of survey variables in the questionnaire that are thought to share similar qualities and are so connected with one another. A rating of 0.70 and above was used to indicate high dependability.

After thorough line-by-line transcript coding of the pretest findings, the KIIs' reliability were tested using an inter-coder reliability test. Transcripts from the KIIs were coded using

separate coding frames. For each of the KIIs, two coding frames were constructed separately, one by the researcher and the other by a competent research assistant. To determine inter-coder reliability, the researcher counted all of the codes from each pair of transcripts evaluated by the two coders using the coding frames. The total number of similar codes assigned to transcript content (agreed coding) was calculated from the total number of codes assigned to transcripts. The percentage of agreement was then calculated, with the numerator being the number of agreed-upon codes and the denominator being the total number of codes allocated. For this investigation, inter-coder reliability scores of 80% or higher were considered adequate.

3.10 Data Collection Methods and Procedures

To collect data for this project, a questionnaire survey and KIIs were undertaken. Using a standardized questionnaire, the study conducted a questionnaire survey with 383 nurses, CHWs, maternity patients, health facility in-charges, and health facility management committee members. Before data collection began, the questionnaires were accompanied by an informed consent form that was willingly completed by the respondent and the investigator. The study questionnaire was self-administered on a single day per health facility using a drop and pick approach.

Key Informant Interviews were carried out among 7 county and sub-county reproductive health coordinators. Because qualitative research is flexible, the researcher adjusted the KII guides based on information gathered during data collection. The KIIs were carried out by the respective researcher with the assistance of an assistant. The KIIs were done on the same day as the questionnaire survey at the appropriate facilities. Participants in KIIs were encouraged to participate in open-ended conversations. All KII sessions were recorded using a digital voice recorder and verbatim transcribed by the researcher. Each KII session lasted roughly 60 minutes on average.

3.11 Data Analysis Techniques and Procedures

Prior to analysis, quantitative data from the questionnaire survey and QoDoS tool were examined for errors, missing data, outliers, and duplicates. IBM SPSS Statistics for Windows, Version 25, was used to clean, code, and process the data. To summarize data on participants' demographic information and specific variables, descriptive analyses were performed, including frequencies, proportions, means, standard deviations, and coefficients of variation. To detect relationships between variables, inferential analyses were performed using the Phi correlation coefficient and binary regression analysis (at a significance level of 0.05). The data were utilized to derive inferences about the impact of PM&E approaches during the initiation, design and planning, and implementation phases on decision making in Mombasa County MNH programs. Tables and figures were used to show the information gathered.

Content analysis was used to assess qualitative data from KIIs. This entailed identifying, labeling, and categorizing main patterns in data, as well as establishing significant linkages. In general, content analysis is used to characterize a phenomenon, in this instance, the application of PM&E methodologies and their impact on decision making in MNH programs in Mombasa County. It helps a researcher to immerse herself in the data, allowing fresh ideas to arise.

3.12 Ethical Considerations

All relevant research clearances were sought from all parties. These included ethical approval from Mount Kenya University's IERC, the NACOSTI, and the Department of Health Services in Mombasa, as well as Medical Superintendents at the health facility and Community Health Extension Workers (CHEWs) at the community health unit levels. These approvals were sought through official requests to local authorities, with approval letters awaiting a response.

Each participant's voluntary involvement in the study was requested through informed consent. Prior to data collection, all participants were given a clear and informative participant informed consent form (appendix 1), and any issues or clarifications raised by the participants were addressed by the researcher. When respondents willingly agreed to participate in the study, they were requested to sign the consent form. Data collection began only when participants had signed the consent form indicating that they fully understood and accepted the parameters of the study. The consent of maternity patients who were below 18 years was sought form the guardian accompanying the patient. Participants were also notified of their ability to withdraw from the research at any time without penalty.

The study's design did not expose participants to any physical, psychological, or socioeconomic dangers. Participants were also informed to ignore any question that caused them distress. Respondents also had the option to pull out at any time if they did not feel comfortable continuing. Furthermore, the volunteers did not get any direct benefits (either monetary or in kind) as a result of their involvement in the study. However, respondents were enlightened about the study's advantages in boosting PM&E and, as a result, decision making in MNH programs throughout the county.

Throughout the study, confidentiality and anonymity were maintained. Participants were guaranteed that their names, as well as the facilities, would be kept private in order to safeguard their identities. Participant logs, which served as the only link between participant names and code numbers, as well as all questionnaires and KII transcripts, were stored in a closed filing cabinet. These files were accessible only to the researcher and the research assistant. The code books that linked the names of the participants to the questionnaire codes, as well as the KII audio recordings, were destroyed once they had been translated and reviewed for correctness. The researcher and the research assistant did not photocopy or remove papers from designated facilities.

The study's findings are submitted to MKU for award of a master's degree in public health and published in a renowned health publication. The findings are also presented to the Mombasa County Department of Health in order to elicit suitable steps for boosting PM&E and decision making in MNH programs in the county. All of the materials reviewed in the study have been properly recognized using APA format citations and references.

CHAPTER FOUR: RESULTS AND DISCUSSIONS

4.1 Introduction

This chapter provides the findings and interpretations on the use of PM&E methodologies and their effect on decision making in Mombasa County MNH programs. IBM SPSS for Windows, version 25, was used to evaluate the obtained data. The results are presented in tables, which are then interpreted. The findings are grouped in accordance with the study's objectives.

4.1.1 Response Rate

The researcher distributed 383 questionnaires to the study respondents out of which 361 questionnaires were returned. After processing the questionnaires, 12 questionnaires were omitted from the analysis because they were incompletely filled. Therefore, 349 questionnaires, representing a response rate of 91%, were used in data analysis. Mugenda and Mugenda (2008), notes that an acceptable response rate should be above 50%. The questionnaire response rate is summarized in Table 4.1. In addition, 6 out of 7 KIIs were conducted.

Table 4.1: Questionnaire Response Rate

Issued Questionnaires	383
Returned questionnaires	361
Analyzed questionnaires	349
**Response Rate**	**91%**

4.1.2 Reliability of Results

Primary data was collected using questionnaires consisting of 5-point Likert scale data sets which were used to measure respondents' opinion towards the underlying constructs in the study. PM&E at the initiation phase was assessed using 3 Likert-type items which yielded a Cronbach's alpha of 0.706; PM&E at the design and planning phase was assessed using 3

Likert-type items which yielded a Cronbach's alpha of 0.755; PM&E at the implementation phase was assessed using 3 Likert-type items which yielded a Cronbach's alpha of 0.793; while quality of decision making was assessed using 44 Likert-type items which yielded a Cronbach's alpha of 0.759. All the Cronbach's alpha test scores were >0.7 indicating that the Likert scales were reliable in representing the underlying constructs in the study. Table 4.2 presents the reliability statistics.

Table 4.2: Reliability Tests

Variable	Cronbach's Alpha	No. of Items
PM&E at the Initiation Phase	.706	3
PM&E at the design and planning phase	.755	3
PM&E at the implementation phase	.793	3
Quality of Decision Making	.759	44

4.2 Descriptive Results

To rate stakeholders' opinions, descriptive statistics such as mean, standard deviation, and coefficient of variation were generated. The width/range of every point in the likert scale was 0.8 [(5-1) ÷ 4]. Therefore, the 5 points in the likert scale were defined by mean ranges including 1 to 1.8, 1.81 to 2.6, 2.61 to 3.4, 3.41 to 4.2, and 4.21 to 5. Coefficients of variation (C.V) were also computed to determine the degree to which individual responses varied from the mean, with C.V>30% considered high. Thus, C.V < 30% implied that the mean represented the collective opinion of the respondents.

4.2.1 Socio-Demographic Information

The researcher gathered some socio-demographic data on the survey respondents in order to understand their composition, qualifications and experience in MNH programs and PM&E. The information collected included the respondents' gender, age, facility level, occupation,

duration of service, training on M&E, and form of training. The results indicated that 210 (60%) were between 26-35 years, 275 (79%) of the respondents were female, while 182 (52%) were CHVs from level 1 health units. Additionally, the respondents 274 (79%) of the respondents had acquired M&E training, out of which 195 (71%) acquired a pre-service training , 75 (27%) through workshops/seminars, and 4 (2%) though on-job training. Table 4.3 presents the demographic results.

Table 4.3: Socio-Demographic Information

Characteristic		N	F	%
What is your gender?	Male	349	74	21.2
	Female		275	78.8
How old are you?	15-25	349	40	11.5
	26-35		210	60.2
	36-45		91	26.1
	46-55		8	2.3
Which level of the health system do you belong to?	Level 1	349	182	52.1
	Level 2		114	32.7
	Level 3		53	15.2
What is your occupation in the health facility or community health unit?	In-Charges	349	36	10.3
	MCM		36	10.3
	Nurses		17	4.9
	CHVs		182	52.1
	Maternity Patients		78	22.3
For how long have you been an in-charge, nurse, management committee member, or a CHW in this health facility/community unit?	Less than 1 year	271	40	14.8
	1-5 years		178	65.7
	6-10 years		46	17.0
	More than 10 years		7	2.6
As a maternity patient, how long have you been seeking MNH services in this facility?	Less than 3 months	78	33	42.3
	3-6 months		26	33.3
	More than 6 months		19	24.4
Are you trained on Monitoring and Evaluation (M&E)?	No	349	75	21.5
	Yes		274	78.5
If you are trained on M&E, where did you acquire the training?	Pre-Service Training	274	195	71.2
	On-Job Training		4	1.5
	Workshops/Seminars		75	27.4

Findings reveal that majority respondents were female which is consistent with the human resource for health (HRH) records at the department of health in Mombasa County which indicates that majority of the healthcare personnel and the CHVs (which constituted the majority of the study's respondents) are female (Mombasa County, 2020). This is also

supported by the fact that all the maternity patients were female. The result also show that more than 80% of those polled were between the ages of 26 and 45 years which is consistent with the working age of majority of the health workers and CHVs (Mombasa County, 2020), and the maternal age of most maternity patients in Mombasa County (Mombasa County, 2018). The results further demonstrates that more than half of those polled were CHVs drawn from level 1 health units (community health level) in Mombasa County. This is consistent with the HRH records from Mombasa County which shows that among the HRH considered in this study, CHVs constitute more than half of the HRH population (Mombasa County, 2020).

The results also show that majority of the in-charges, MCM and CHVs had worked in their respective stations for more than 1 year but less than five years. Majority of the maternity patients had sought services in the respective health facilities for more than 3 months. This shows that these categories of respondents, who make up the management, service providers and beneficiaries in MNH programs, had reliable experience with the utilization, or not, of PM&E in MNH programs in Mombasa County. This makes the information derived from them more reliable in answering the research questions.

The results further indicate that the respondents, who are important stakeholders in MNH programs, were trained on PM&E, with majority obtaining their training during their formal education. This implies that these stakeholders have the knowledge and skills to be able to be involved as active participants in MNH programs. However, it is also worth to note that continuous education through on-job training and workshops/seminars on PM&E is essential in ensuring that PM&E is effectively utilized in the MNH interventions. Kananura et al. (2017) identified the need to improve PM&E skills in order to expand the spaces for decision-making for both key planners and implementers.

4.2.2 Utilization of PM&E in MNH Programs

The utilization of PM&E approaches in MNH programs was measured using a set of 4 likert scale items rated on a 5-point scale of evaluation (1 to 1.8 means strongly disagree, 1.81 to 2.6 means disagree, 2.61 to 3.4 means neutral, 3.41 to 4.2 means agree, while 4.21 to 5 means strongly agree). Respondents neither agreed nor disagreed that primary stakeholders are considered as active participants in the initiation, design and implementation of MNH programs ($\bar{X}$ = 3.30, σ = 0.831, CV = 25%), and that the capacity of primary stakeholders in PM&E (to collect data, analyze, reflect and take action) is enhanced through training and continuous mentorship ($\bar{X}$ = 2.94, σ = .835, CV = 28%). However, the respondents agreed that joint information reviews and learning among stakeholders is fostered at various levels of PM&E ($\bar{X}$ = 4.45, σ = 0.579, CV = 13%), and that stakeholders are encouraged to be accountable and responsible in taking corrective action(s) based of PM&E information ($\bar{X}$ = 4.36, σ = 0.740, CV = 17%). Aggregated results indicate that PM&E approaches were utilized in MNH programs in Mombasa County ($\bar{X}$ = 4.06, σ = 0.636, CV = 16%). The aggregated CV<30 shows that the mean aggregates represented the respondents' collective opinions. Findings are shown in Table 4.4.

Table 4.4: Utilization of PM&E in MNH Programs in Mombasa County

Utilization of PM&E Approaches	N	Min	Max	Mean	Std. Dev.	C.V (%)
Primary stakeholders are considered as active participants in the initiation, design and design, and implementation of MNH programs	349	1	5	3.30	.831	25.2
The capacity of primary stakeholders in PM&E (to collect data, analyze, reflect and take action) is enhanced through training and continuous mentorship.	349	1	4	2.94	.835	28.4
Joint information reviews and learning among stakeholders is fostered at various levels of PM&E	349	2	5	4.45	.579	13.0
Stakeholders are encouraged to be accountable and responsible in taking corrective action(s) based of PM&E information.	349	2	5	4.36	.740	17.0
**Utilization of PM&E approaches**	**349**	**1**	**5**	**4.06**	**.636**	**16.0**

Findings from the KIIs also indicated that PM&E was utilized in MNH programs in Mombasa County. This is shown by the responses from the respondents as indicated below;

> *"I have to emphasize that we have a lot of expertise with M&E techniques in MNH programs. Various stakeholders are involved from the point of data collection to the use of data for decision-making...Staff are sufficiently trained since we have M&E process guides on surveillance rules that are updated on a regular basis..."* KII 3

Another KII respondent indicated that;

> *"We have effectively adopted M&E in various MNH programs in assessing the progress against expected results, to spot bottlenecks in implementation and to highlight the positive or negative effects of the various interventions."* KII 5

The results show that PM&E approaches were utilized to some extent in MNH programs in Mombasa County. Sifunjo (2019), Kananura et al. (2017) and Kajaga (2016) concurred with the study's findings revealing that PM&E approaches were utilized in the respective programs in Kajiado County, eastern Uganda, and Gulu District. However, the findings differ from those of Karanja (2016) which showed that stakeholders were not engaged in all the phases of the CDF project in Dagoretti South Sub-County.

As much as the findings indicate that stakeholders are engaged in information reviews and decision making, the department of health in Mombasa County needs to recognize and build the capacity of MNH staff and the beneficiary communities to become active participants in monitoring and evaluation of the different stages of MNH programs. This would broaden the concept of accountability to include not just whether the department and various health institutions are meeting the terms of their financing, but also whether

they are meeting the needs and aspirations of the communities they serve. As Karanja (2016) posits, the primary goal of PM&E is to provide data to beneficiaries and program managers in order for them to review if project objectives were met and how the appropriate authorities used resources to improve project management and make key choices. Therefore, the utilization of PM&E approaches in MNH programs will ensure that the various interventions generate value for money and address the underlying needs of the beneficiaries.

4.2.3 Quality of Decision Making in MNH Programs

The quality of decision making in MNH programs at both the organizational level and individual level was assessed using modified QoDoS, based on four indicators. Two of the indicators, decision-making approach and decision-making culture, measured the quality of decision making at the organizational level, while decision-making competence and decision-making style measured the quality of decision making at the individual level. The four indicators were measured using sets of 5-point likert scales (1 to 1.8 – Not at all; 1.81 to 2.6 – Sometimes; 2.61 to 3.4 – Frequently; 3.41 to 4.2 – Often; 4.21 to 5 – Always).

Decision making approach and decision making culture in MNH programs were measured using a set of 12 and 8 likert type items respectively. The sets of likert items were aggregated to measure the frequency with which decision making practices at the health facilities constituted effective decision making approach and decision making culture respectively. Results in Table 4.5 show that decision making practices at the health facilities often constituted effective approach to decision making ($\bar{X}$ = 3.89, σ = 0.321, CV = 8%). The findings also indicate that decision making practices at the health facilities sometimes constituted effective culture to decision making ($\bar{X}$ = 2.05, σ = 0.485, CV = 24%). Additionally, the scores for decision making approach and decision making culture were

aggregated to measure the frequency with which decision making practices at the organization level constituted quality decision making. Results indicate that decision making practices at the organization level frequently constituted quality decision making ($\bar{X}$ = 3.00, σ = 0.240, CV = 8%).

Decision making competence and decision making style in MNH programs were measured using a set of 14 and 10 likert type items respectively. The sets of likert items were aggregated to measure the frequency with which decision making practices of individuals at the health facilities constituted competent decision making and effective style of decision making respectively. Results in Table 4.5 show that decision making practices of individuals at the health facilities often constituted competent decision making ($\bar{X}$ = 3.75, σ = 0.454, CV = 12%). The findings also indicate that decision making practices of individuals at the health facilities sometimes constituted effective style to decision making ($\bar{X}$ = 1.99, σ = 0.273, CV = 14%). The scores for decision making competence and decision making style were further aggregated to measure the frequency with which decision making practices at the individual level constituted quality decision making. Results indicate that decision making practices at the individual level frequently constituted quality decision making ($\bar{X}$ = 2.94, σ = 0.287, CV = 10%).

The scores for decision making level at organizational level and individual level were then aggregated to measure the frequency with which decision making practices at the health facilities constituted quality decision making. Results in Table 4.5 indicate that decision making practices at the health facilities frequently constituted quality decision making ($\bar{X}$ = 2.95, σ = 0.223, CV = 8%). The individual scores did not vary significantly from the average scores (C.V<30%) indicating the mean scores were representative of the respondent's opinion.

Table 4.5: Quality of Decision Making in MNH Programs in Mombasa County

Indicators	N	Min	Max	Mean	Std. Dev.	C.V (%)
**Organization Level**						
Decision Making Approach	349	3	5	3.89	.321	8.3
Decision Making at Culture	349	1	3	2.05	.485	23.7
**Decision Making at the Organizational Level**	**349**	**2**	**4**	**3.00**	**.240**	**8.0**
**Individual Level**						
Decision Making Competence	349	2	4	3.75	.454	12.1
Decision Making Style	349	1	3	1.99	.273	13.7
**Decision Making at the Individual Level**	**349**	**2**	**4**	**2.94**	**.287**	**9.8**
Quality of Decision Making in MNH Programs	349	2	4	2.95	.223	7.6

The results on the assessment of the four indicators of decision making in MNH programs showed that the decision-making practices at both the individual and organizational level in health facilities in Mombasa County were generally favorable practices. However, there were a few practices that needed improvement, for instance, providing training to stakeholders in the science of decision making. Training on decision making is essential in providing and enhancing the management and leadership skills needed at every stage of MNH programs. It will encourage good decision making, which is essential for attaining goals and providing the necessary results. Kananura et al. (2017) identified the need to improve PM&E skills in order to expand the spaces for decision-making for both key planners and implementers.

4.2.4 Utilization of PM&E Approaches at the MNH Programs' Initiation Phase

Utilization of PM&E approaches at the initiation phase in MNH programs was measured using a set of 3 likert type items rated on a 5-point likert scale (1 to 1.8 – Not at all; 1.81 to 2.6 – Sometimes; 2.61 to 3.4 – Frequently; 3.41 to 4.2 – Often; 4.21 to 5 – Always). The respondents indicated that at the MNH programs' initiation phase, participatory analysis of objectives was often conducted ($\bar{X} = 3.50$, $\sigma = 0.628$, CV = 18%). However, the respondents

opinion was divided (CV>30%) on the frequency with which participatory baseline assessment ($\bar{X}$ = 2.96, σ = 0.901, CV = 30%) and participatory needs assessment ($\bar{X}$ = 2.24, σ = 0.931, CV = 42%) were conducted. The scores for the 3 approaches were further aggregated to measure the frequency with which PM&E approaches were utilized at the MNH programs' initiation phase. Results indicated that PM&E approaches were frequently utilized at the MNH programs' initiation phase ($\bar{X}$ = 2.91, σ = 0.648, CV = 22%). Results are shown in Table 4.6.

Table 4.6: Utilization of PM&E Approaches at the Initiation Phase in MNH Programs in Mombasa County

PM&E at the Initiation Phase	N	Min	Max	Mean	Std. Dev.	C.V (%)
Participatory needs assessment	349	1	4	2.24	.931	41.6
Participatory baseline assessment	349	1	5	2.96	.901	30.4
Participatory analysis of objectives	349	1	5	3.50	.628	17.9
**Utilization of PM&E Approaches at the Initiation Phase**	**349**	**1**	**5**	**2.91**	**0.648**	**22.3**

Findings from the KIIs also showed that PM&E approaches were utilized at the initiation phase of MNH programs in Mombasa County. Below is a response from one of the respondents;

> *"…Under the youth friendly sexual and reproductive health initiative in the county, focus group discussions with the youth are conducted within youth friendly clinics, e.g. Mwembe Tayari and Junda, to understand the sexual and reproductive health needs of the youth population in the county, in order to come up with services that respond to their needs. The overall aim of the focus group discussions is to understand the sexual and reproductive health needs of the youth from their own perspective rather than from the providers' view point. This*

approach has helped in increasing the number of youth seeking sexual and reproductive health services in the youth friendly clinics…” KII 6

The findings show that the utilization of PM&E approaches at the initiation phase of MNH programs in Mombasa County was moderate. This finding is inconsistent with the findings of Sifunjo (2019) which established that stakeholders in MNH programs in Kajiado county, Kajiado North constituency were highly involved in needs assessment, project identification, objective analysis among other participatory approaches at the initiation phase. Mainstreaming participation in conducting needs assessment, baseline assessment, and analysis of objectives at the initiation phase of MNH programs will help ensure the long-term viability of maternal health programmes and that outcomes respond to beneficiaries' needs and expectations. According to Sifunjo (2019), participatory approaches at the initiation phase results in the long-term viability of maternal health programmes and improved success of maternal health programs.

4.2.5 Utilization of PM&E at the Design and Planning Phase in MNH Programs

Utilization of PM&E approaches at the design and planning phase in MNH programs was measured using a set of 3 likert type items rated on a 5-point likert scale (1 to 1.8 – Not at all; 1.81 to 2.6 – Sometimes; 2.61 to 3.4 – Frequently; 3.41 to 4.2 – Often; 4.21 to 5 – Always). The respondents indicated that participatory feasibility analysis ($\bar{X}$ = 3.50, σ = 0.628, CV = 18%) and participatory SWOT analysis ($\bar{X}$ = 3.88, σ = 0.458, CV = 12%) were often conducted, while participatory risk assessment was frequently conducted ($\bar{X}$ = 3.06, σ = 0.798, CV = 26%). The scores for the 3 approaches were further aggregated to measure the frequency with which PM&E approaches were utilized at the MNH programs' design and planning phase. Results indicate that PM&E approaches were often utilized at the MNH programs' planning and design phase ($\bar{X}$ = 3.59, σ = 0.598, CV = 17%). The individual

scores yielded CV<30% showing that the mean aggregates represented respondents' collective opinions. Results are shown in Table 4.7.

Table 4.7: Utilization of PM&E Approaches at the Design & Planning Phase in MNH Programs in Mombasa County

PM&E at the Design & Planning Phase	N	Min	Max	Mean	Std. Dev.	C.V (%)
Participatory feasibility analysis	349	1	5	3.68	.664	18.0
Participatory SWOT analysis	349	2	5	3.88	.458	11.8
Participatory risk assessment	349	2	5	3.06	.798	26.1
**Utilization of PM&E Approaches at the Design & Planning Phase**	**349**	**1**	**5**	**3.59**	**0.598**	**16.7**

Findings from the KIIs also revealed that PM&E approaches were utilized at the design and planning phase of MNH programs in Mombasa County. The following are responses from some respondents;

> *"Yes, we use SWOT analysis in a collaborative and participatory manner in the development of plans to define the framework of the MNH programs' main threats associated with weaknesses and significant opportunities linked to our strengths...Participants are asked to collaborate with ideas, hints, and creative proposals to find the best common strategies for valuing and implementing the MNH programs."* KII 4

Another respondent noted;

> *"We organize for Workshops where we discuss various plans before they are implemented…some of the activities that is done during the planning workshops include feasibility analysis, risk assessment, budget analysis etc….Staff from different departments are involved…a number of stakeholders, especially members of the health facility management committees, are involved in the workshops."* KII 2

The results show that PM&E approaches at the design and planning phase of MNH programs in Mombasa County were favorably utilized. Kananura et al. (2017) agreed with the study's findings establishing that during the design phase of the MNH program in eastern Uganda, focus group discussions and stakeholder meetings with local members of the communities were conducted. According to Kananura et al. (2017) PM&E techniques were beneficial at the design stage for detecting major local challenges and potential home-grown solutions, as well as shaping the activities that were later executed. Kajaga (2016) also concurred with the findings establishing that stakeholders in USAID-SAFE program in Gulu District were involved in the design and planning phase through beneficiaries' participation in planning, defining indicators, priorities, and targets, establishing objectives, identifying problems, and offering solutions to obstacles. However, the findings differ with the findings of Karanja (2016) which revealed that stakeholders were not engaged during the panning phase of CDF projects in Dagoretti South Sub-County.

4.2.6 Utilization of PM&E at the Implementation Phase in MNH Programs

Utilization of PM&E approaches at the implementation phase in MNH programs was measured using a set of 3 likert type items rated on a 5-point likert scale (1 to 1.8 – Not at all; 1.81 to 2.6 – Sometimes; 2.61 to 3.4 – Frequently; 3.41 to 4.2 – Often; 4.21 to 5 – Always). The respondents indicated that Supportive supervision ($\bar{X}$ = 3.95, σ = 0.356, CV = 9%), Participatory performance reviews ($\bar{X}$ = 3.81, σ = 0.581, CV = 15%) and Participatory desk reviews ($\bar{X}$ = 3.96, σ = 0.373, CV = 9%) were often conducted. The scores for the 3 approaches were further aggregated to measure the frequency with which PM&E approaches were utilized at the MNH programs' implementation phase. Results indicate that PM&E approaches were often utilized at the MNH programs' implementation ($\bar{X}$ = 3.59, σ = 0.598, CV = 17%). The individual scores yielded CV<30% showing that the mean aggregates represented respondents' collective opinions. Results are shown in Table 4.8.

Table 4.8: Utilization of PM&E Approaches at the Implementation Phase in MNH Programs in Mombasa County

PM&E at the Implementation Phase	N	Min	Max	Mean	Std. Dev.	C.V (%)
Supportive supervision	349	2	5	3.95	.356	9.0
Participatory performance reviews	349	2	5	3.81	.581	15.2
Participatory desk reviews	349	2	5	3.96	.373	9.4
**Utilization of PM&E Approaches at the Implementation Phase**	**349**	**1**	**5**	**3.80**	**0.503**	**13.2**

Results from the KIIs also revealed that PM&E approaches were utilized at the implementation phase of MNH programs in Mombasa County. Below are responses from some respondents;

> *"We make quarterly supporting monitoring visits to the health institutions....Supportive supervision visits include a variety of tasks, including observation of all departments, evaluation of registers and reports, training, and follow-up on concerns noted during the last visit; nevertheless, the majority of the visit is devoted to data verification."* KII 1

Another KII respondent noted that;

> *"Supportive supervision begins with planning... when we plan, we are increasing the capacity of the supervisory teams. So, to do this, we collaborate and arrange for supporting supervision logistically, in terms of people, checklists, and so on."* KII 6

The results show that PM&E approaches at the implementation phase of MNH programs were highly utilized. Kananura et al. (2017) concurred with this finding establishing that support supervision and mentorship visits, quarterly review meetings and midterm surveys were conducted involving various key stakeholders including the community members, health workers, and sub-county and district health teams. Kananura et al. (2017) found that a mix of

participatory M&E methodologies and stakeholder input was extremely effective in tracking progress and spotting emergent implementation problems, which aided in enabling planning and decision-making throughout the execution phase. Kajaga (2016) also supported the findings showing that during the implementation phase of USAID-SAFE program in Gulu District, PM&E approaches were utilized to a great extent. According to Kajaga (2016), the involvement of stakeholders through the utilization of the PM&E approaches promotes the execution of effective interventions, and dealing with evolving issues during implementation. However, the findings differ with the findings of Karanja (2016) which revealed that stakeholders were not engaged during the implementation phase of CDF projects in Dagoretti South Sub-County.

4.3 Correlation Analysis

In order to assess the relationships between variables, the 5-point Likert scale data sets from the questionnaire were combined into single composite variables by computing the mean scores of the Likert-type items for each Likert scale data set. The composite scores would be more manageable and reliable measures of the underlying study constructs compared to the single Likert-type items. The composite variables (CV) created by reducing the data include, PM&E approaches at the initiation phase; PM&E approaches at the design and planning phase; PM&E approaches at the implementation phase; and quality of decision making.

Additionally, in order to improve the validity of the model, the 5 category response data sets, were merged into 2 category response data sets, rarely and often, using the following formula;

Max = Highest Score (HS) = 5

Min = Lowest Score (LS) = 1

Range = HS - LS = 4

No. of Categories = Two = 2

Interval = Range ÷ No. of Categories = 4 ÷ 2 = 2

Rarely (Category_1) = Min through (Min + Interval) = 1.00 – 3.00

Often (Category_2) = (Max - Interval) through Max = 3.01 – 5.00

The composite scores obtained by combining the Likert-type items were then recoded based on the two category response data sets. Thus, all mean scores falling between 1.00 through 3.00 were recoded to 1 (rarely), while all mean scores falling between 3.01 through 5.00 were recoded to 2 (often).

The researcher conducted a Phi Coefficient test, at a significance level of 0.05, to determine the strength of association between variables. The results indicated that PM&E approaches at the initiation phase ($\phi = 0.164$, $p < 0.05$), PM&E approaches at the design and planning phase ($\phi = 0.203$, $p > 0.05$), and PM&E approaches at the implementation phase ($\phi = 0.199$, $p < 0.05$) had a weak positive relationship with improvement in data management. This signifies that increased utilization of PM&E approaches at the initiation, design and planning, and implementation phases of MNH programs in Mombasa County would be accompanied by improvement in quality of decision making in MNH programs in Mombasa County. Results are shown in Table 4.9.

Table 4.9: Phi Correlation Coefficient

		**Quality of Decision Making**		**Phi Coefficient**		
		**Rarely**	**Often**	Value	N	Sig.
**PM&E Approaches at the Initiation Phase**	Rarely	117	136	.164	349	.002
	Often	27	69			
**PM&E Approaches at the Design & Planning Phase**	Rarely	26	11	.203	349	.000
	Often	118	194			
**PM&E Approaches at the Implementation Phase**	Rarely	15	3	.199	349	.000
	Often	129	202			

4.4 Influence of Utilization of PM&E Approaches on Decision-Making

The degree of influence of PM&E approaches at the initiation, design & planning, and implementation phases on decision making in MNH programs in Mombasa County was measured using binary logistic regression analysis at 0.05 significance level. The last response category was set as the reference group. Results are presented in the following sub-sections.

4.4.1 Goodness-of-fit Test

The Hosmer-Lemeshow test was employed at a significance level of 0.05 to see if the predicted probability differed from the actual probabilities in a way that the binomial distribution did not predict. The Hosmer-Lemeshow statistic indicates a poor fit if the significance value is less than 0.05. Table 4.10 shows that the model effectively matches the data. As a result, there is no distinction between the observed and anticipated models.

Table 4.10: Hosmer and Lemeshow Test

Step	Chi-square	df	Sig.
1	.881	2	.644

Table 4.11 further demonstrates that no difference exists between the observed and anticipated models. Both values are close to one another, indicating that the model adequately fits the data.

Table 4.11: Contingency Table for Hosmer-Lemeshow Test

		Quality of Decision Making = No Improvement		Quality of Decision Making = Improvement		Total
		Observed	Expected	Observed	Expected	
Step 1	1	35	34.955	13	13.045	48
	2	1	.535	0	.465	1
	3	82	82.045	123	122.955	205
	4	26	26.465	69	68.535	95

4.4.2 Classification of Cases

The study also assessed whether the binomial logistic regression model, correctly predicted cases from the independent variables. This was done by assessing the effectiveness of the predicted classification against the actual classification. The results indicate that the model correctly classified 65.3% of cases. Results are shown in Table 4.12.

Table 4.12: Classification Table

Observed			Predicted		
			Quality of Decision Making		Percentage Correct
			No Improvement	Improvement	
**Step 1**	**Quality of Decision Making**	**No Improvement**	36	108	25.0
		**Improvement**	13	192	93.7
	**Overall Percentage**				65.3

a. The cut value is .500

4.4.3 Model Summary

In order to understand how much variation in the outcome variable was explained by the model, the study used Nagelkerke R2. The results indicated that the model explained 20.9% of the variance in quality of decision making in MNH programs in Mombasa County. Results are shown in Table 4.13.

Table 4.13: Model Summary

Step	-2 Log likelihood	Cox & Snell R Square	Nagelkerke R Square
1	413.731[a]	.181	.209

a. Estimation terminated at iteration number 4 because parameter estimates changed by less than .001.

The logistic regression results in Table 4.14 show that the odds of a health facility making quality decisions in MNH programs are 1.728 times higher when PM&E approaches are utilized at the initiation phase than when PM&E approaches are rarely utilized, with a 95% CI

of 1.024 to 2.917. This implies that utilization of PM&E approaches at the initiation phase of MNH programs was a significant predictor of quality decision making. Therefore, health facilities that utilize PM&E approaches at the initiation phase of MNH programs have a high likelihood of making quality decisions. This is supported by the findings of Sifunjo (2019) who examined the impact of participatory monitoring and evaluation on maternal health programs in Kajiado county, Kajiado North constituency. The study found that participatory project identification resulted in the long-term viability of maternal health programmes. The study also found that stakeholder engagement in vision, mission, and target setting improved the success of maternal health programs. The study also discovered that identifying and involving stakeholders has a beneficial impact on the performance of maternal health programs.

The results also show that the odds of a health facility making quality decisions in MNH programs are 2.977 times higher when PM&E approaches are utilized at the design and planning phase than when PM&E approaches are rarely utilized, with a 95% CI of 1.380 to 6.424. This suggests that utilization of PM&E approaches at the design and planning phase of MNH programs was a significant predictor of quality decision making. Hence, health facilities that utilize PM&E approaches at the design and planning phase of MNH programs have a high probability of making quality decisions. Kananura et al. (2017) who investigated how participatory M&E techniques aided in the identification of critical design and implementation difficulties, as well as how they affected decision-making among stakeholders in eastern Uganda, supported the study's findings. The author established that the PM&E techniques were beneficial at the design stage for detecting major local challenges and potential home-grown solutions, as well as shaping the activities that were later executed.

Additionally, the results show that the odds of a health facility making quality decisions in MNH programs are 5.665 times higher when PM&E approaches are utilized at the

implementation phase than when PM&E approaches are rarely utilized, with a 95% CI of 1.569 to 20.459. This implies that utilization of PM&E approaches at the implementation phase of MNH programs was a significant predictor of quality decision making. Thus, health facilities that utilize PM&E approaches at the design and planning phase of MNH programs have a high chances of making quality decisions. The findings support the findings of Kananura et al. (2017) who established that at the implementation phase, the PM&E approaches provided factual data that formed the basis for decision-making and assisted in the identification of evolving issues.

Table 4.14: Binary Logistic Regression

Independent Variables	B	S.E.	Wald	df	Sig.	Exp(B)	95% C.I. for EXP(B)	
							Lower	Upper
PM&E at the Initiation Phase	.547	.267	4.193	1	.041	1.728	1.024	2.917
PM&E at the Design & Planning Phase	1.091	.392	7.730	1	.005	2.977	1.380	6.424
PM&E at the Implementation Phase	1.734	.655	7.007	1	.008	5.665	1.569	20.459
Constant	-2.421	.716	11.422	1	.001	.089		

a. Variable(s) entered on step 1: PM&E at the Initiation Phase, PM&E at the Design & Planning Phase, PM&E at the Implementation Phase.

CHAPTER FIVE

SUMMARY OF FINDINGS, CONCLUSIONS AND RECOMMENDATIONS

5.1 Introduction

This chapter summarizes the study's findings, conclusions, recommendations, and areas for future research.

5.2 Summary of Findings

The general objective was to assess the utilization of PM&E approaches and its influence on decision making in MNH programs in Mombasa County. The study employed descriptive cross-sectional research and collected data from 349 survey respondents and 7 key informant interviewees drawn from a target population comprising of 1500 CHWs, 120 nurses, 570 maternity patients, 36 health facility in-charges and 288 health facility management committee members in 36 levels 2 and 3 public health facilities and community health units in the six sub-counties (Mombasa County, 2020). Data were analysed using both descriptive and inferential statistics. Association between the variables in the study was assessed using the phi correlation coefficient and binary logistic regression at a significance level of 0.05. Descriptive analysis findings showed that quality decision making was frequently made in MNH programs in Mombasa County. Furthermore, the results of the regression analysis revealed that PM&E approaches at the MNH programs' initiation, design and planning, and implementation phases were a significant determinant of quality of decision making in MNH programs in Mombasa County.

The first specific objective was to assess the influence of utilization of PM&E approaches at the program initiation phase on the quality of decision making in MNH programs in Mombasa County. The findings of the descriptive analysis revealed that participatory needs assessment and participatory baseline assessment were frequently conducted, while participatory analysis

of objectives was often conducted in MNH programs. Correlation analysis revealed that the utilization of PM&E approaches at the initiation phase of MNH programs was positively associated with quality decision making. Additionally, the results of the regression analysis revealed that quality decision making was more likely to occur when PM&E approaches were utilized at the initiation phase of MNH programs than when the PM&E approaches were not utilized.

The second specific objective was to assess the influence of utilization of PM&E approaches at the program design and planning phase on the quality of decision making in MNH programs in Mombasa County. The findings of the descriptive analysis revealed that participatory feasibility analysis and participatory SWOT analysis were often conducted, while participatory risk assessment was frequently conducted in MNH programs. Correlation analysis revealed that the utilization of PM&E approaches at the design and planning phase of MNH programs was positively associated with quality decision making. In addition, the results of the regression analysis revealed that the likelihood of quality decision making occurring was higher when PM&E approaches were utilized at the design and planning phase of MNH programs than when the PM&E approaches were not utilized.

The third specific objective was to evaluate the influence of utilization of PM&E approaches at the program implementation phase on the quality of decision making in MNH programs in Mombasa County. The findings of the descriptive analysis revealed that supportive supervision, participatory performance reviews, and participatory desk reviews were often conducted in MNH programs. Correlation analysis revealed that the utilization of PM&E approaches at the implementation phase of MNH programs was positively associated with quality decision making. Furthermore, the results of the regression analysis revealed that the odds of quality decision making occurring were higher when PM&E approaches were utilized

at the implementation phase of MNH programs than when the PM&E approaches were not utilized.

5.3 Conclusions

The study concludes that utilization of PM&E approaches at the initiation phase had a significant effect on quality of decision making in MNH programs in Mombasa County. Quality decision making was more likely to occur in MNH programs when PM&E approaches were utilized at the initiation phase of MNH programs than when the PM&E approaches were not utilized.

The study also concludes that utilization of PM&E approaches at the design and planning phase had a significant effect on quality of decision making in MNH programs in Mombasa County. Quality decision making was more likely to occur in MNH programs when PM&E approaches were utilized at the design and planning phase of MNH programs than when the PM&E approaches were not utilized.

The study further concludes that utilization of PM&E approaches at the implementation phase had a significant effect on quality of decision making in MNH programs in Mombasa County. Quality decision making was more likely to occur in MNH programs when PM&E approaches were utilized at the implementation phase of MNH programs than when the PM&E approaches were not utilized.

5.4 Recommendations

On the basis of the research findings, the department of health in Mombasa County should adopt PM&E approaches in MNH programs' initiation, design and planning, and implementation phases in order to benefit from rational, timely, and relevant information for decision-making, and thus improve on delivery of MNH services.

The researcher also recommends that the department of health in Mombasa County should develop sound policies to support decision-makers, budget planners, health service providers, and community stakeholders (such as community health workers) to adopt PM&E in MNH programs in order to facilitate effective management of health systems, resource allocation based on need, and effective service delivery.

The researcher also recommends that the department of health in Mombasa County should invest heavily on knowledge acquisition for its managers and staff on how to effectively adopt and implement different PM&E approaches in MNH programs.

5.5 Suggestions for Further Research

Future studies should use a longitudinal research design to generate findings that support more valid conclusions about the cause-and-effect relationship between the variables in the study.

According to the findings, utilization of PM&E approaches at the initiation, design and planning, and implementation phases did not fully explain the variation in quality of decision making in MNH programs in Mombasa County. As a result, additional research should be conducted to focus on other factors influencing Quality of decision making in MNH programs in the county.

A comparable study should be conducted in other counties in order to relate findings and generate more knowledge on PM&E approaches associated with improved quality decision making in the health sector and thus assist policy makers, managers, staff, and other stakeholders in adopting effective strategies to facilitate effective management of health systems, resource allocation based on need, and taking responsibility for keeping health-care promises.

REFERENCES

Bell, S., & In Aggleton, P. (2016). *Monitoring and evaluation in health and social development: Interpretive and ethnographic perspectives*. New York: Routledge, Taylor & Francis Group.

Bujar, M., Donelan, R., McAuslane, N., Walker, S., & Salek, S. (2016a). Assessing the quality of decision making in the development and regulatory review of medicines: identifying biases and best practices. *Ther. Innov. Reg Sci.* 51, 250–256. doi: 10.1177/2168479016662681

Bujar, M., McAuslane, N., Salek, S., & Walker, S. (2016b). Quality of regulatory decision-making practices: issues facing companies and agencies. *Ther. Innov. Reg. Sci.* 50, 487–495. doi: 10.1177/2168479016628573

Bujar, M., McAuslane, N., Walker, S.R. & Salek, S. (2017) Evaluating Quality of Decision-Making Processes in Medicines' Development, Regulatory Review, and Health Technology Assessment: A Systematic Review of the Literature. *Front. Pharmacol.* 8:189. doi: 10.3389/fphar.2017.00189

Chen H. (2012) Theory-driven evaluation: Conceptual framework, application and advancement. In: Strobl R., Lobermeier O., Heitmeyer W. (eds) *Evaluation von Programmen und Projekten für eine demokratische Kultur*. Wiesbaden: Springer VS.

Chen, H. (1990). *Theory-driven evaluations*. Newbury Park, Calif: Sage Publications.

Chen, H. (2005). *Practical program evaluation: Assessing and improving planning, implementation, and effectiveness*. Thousand Oaks, Calif: Sage.

Daniels, N. (2016). Resource Allocation and Priority Setting. In: H. Barrett D, W. Ortmann L, Dawson A, et al. (editors). *Public Health Ethics: Cases Spanning the Globe*. Cham

(CH): Springer https://www.ncbi.nlm.nih.gov/books/NBK435786/ doi: 10.1007/978-3-319-23847-0_3

Donelan, R., Walker, S., & Salek, S. (2015). Factors influencing quality decision making: regulatory and pharmaceutical industry perspectives. *Pharmacoepidemiol. Drug Saf.* 24, 319–328. doi: 10.1002/pds.3752

Donelan, R., Walker, S., & Salek, S. (2016). The development and validation of a generic instrument, QoDoS, for assessing the quality of decision making. *Front Pharmacol.* 7:180. doi: 10.3389/fphar.2016.00180

Fetterman, D.M. (1994). Empowerment evaluation. *Evaluation Practice*, 15(1), 1-15.

Fetterman, D.M. (2015). Empowerment Evaluation and Action Research: A Convergence of Values, Principles, and Purpose. In Bradbury, H. (ed.) *The Handbook of Action Research*. Thousand Oaks, CA: Sage.

Funnell, S. C., & Rogers, P. J. (2011). *Purposeful program theory: Effective use of theories of change and logic models*. San Francisco, CA: Jossey-Bass.

Glandon, D., Paina, L., Alonge, O., Peters, D.H., & Bennett, S. (2017). 10 Best resources for community engagement in implementation research. *Health Policy and Planning*, *32*(10), 1457–1465. https://doi.org/10.1093/heapol/czx123

Hilhorst, T. & Guijt, I.M. (2006). Participatory monitoring and evaluation: a process to support governance and empowerment at the local level: a guidance paper. *BMC Infectious Diseases*.

Holvoet N, & Inberg L. (2014). Taking stock of monitoring and evaluation systems in the health sector: Findings from Rwanda and Uganda. *Health Policy and Planning*, *29*, 506–16.

Jamaal, N. (2018). Effects of participatory monitoring and evaluation on project performance at Kenya Marine and Fisheries Research Institute, Mombasa, Kenya. *International Academic Journal of Information Sciences and Project Management*, *3*(1), 1-15.

Johnson, B.R., & Christensen, L.B. (2017). *Educational research: Quantitative, qualitative, and mixed approaches*. Los Angeles: SAGE.

Kajaga, R. (2016). *Participatory Monitoring and Evaluation (PM&E) and Service Delivery in Uganda: A Case of USAID-SAFE Program in Gulu District*, Unpublished MSc Thesis. Kampala: Uganda Management Institute.

Kananura, R.M., Ekirapa-Kiracho, E., Paina, L., Bumba, A., Mulekwa, G., Nakiganda-Busiku, D., … Peters, D.H. (2017). Participatory monitoring and evaluation approaches that influence decision-making: lessons from a maternal and newborn study in Eastern Uganda. *Health Research Policy and Systems*, *15*(Suppl 2), 107. doi: 10.1186/s12961-017-0274-9. PMID: 29297410; PMCID: PMC5751403.

Karanja, G.N. (2016). *Assessment of the use of participatory monitoring and evaluation approach: a case of constituency development fund projects in Dagoretti south sub-county Nairobi, Kenya*, Unpublished MA Thesis. Nairobi: University of Nairobi.

Kathongo, S. M. (2018). Influence of Participatory Monitoring and Evaluation on Performance of Public Secondary Schools Projects in Mutomo Sub-County, Kenya. *International Journal of Scientific Research and Management*, 6(03), EL–2018. https://doi.org/10.18535/ijsrm/v6i3.el06

Kotch, J. (2013). *Maternal and child health: Programs, problems, and policy in public health*. Sudbury, Mass: Jones & Bartlett Learning.

Kothari C. R., & Gaurav, G. (2014). *Research Methodology: Methods and Techniques*, (3rd ed.). New Delhi: New Age International (P) Limited.

Lubita, Mubati, & Mulonda, (2017). The Importance and Limitations of Participation in Development Projects and Programmes. *European Scientific Journal*, *13*(5), 238-251.

Luutu, P.P. (2016). *Participatory evaluation and utilisation of evaluation results in Share an Opportunity (SAO) Uganda*, Unpublished MSc Thesis. Kampala: Uganda Technology and Management University.

Maldonado, L. Y., Bone, J., Scanlon, M. L., Anusu, G., Chelagat, S., Jumah, A., Ikemeri, J. E., Songok, J. J., Christoffersen-Deb, A., & Ruhl, L. J. (2020). Improving maternal, newborn and child health outcomes through a community-based women's health education program: a cluster randomised controlled trial in western Kenya. *BMJ Global Health*, *5*(12), e003370. https://doi.org/10.1136/bmjgh-2020-003370

Mohammed, S., Alhassan, B.M., & Kanlisi, K.S. (2018). An Assessment of Stakeholder Participation in Monitoring and Evaluation of District Assembly Projects and Programmes in the Savelugu-Nanton Municipality Assembly, Ghana. *Ghana Journal of Development Studies*, *15*(1), 173-195.

Mombasa County Government. (2018). *Second Health Strategic and Investment Plan (CHSIP II) 2018 –2022: A Healthy and Productive Community*. Mombasa: Mombasa County Government, Department of Health Services.

Mombasa County Government. (2020). *Mombasa County Human Resources for Health*. Mombasa: Department of Health Mombasa County.

Mulwa, F. W. (2008). *Participatory monitoring and evaluation of community projects: Community based project monitoring, qualitative impact assessment, and people-friendly evaluation methods*. Nairobi: Paulines Pub. Africa.

Nayak, J. K. & Singh, P. (2015). *Fundamentals of Research Methodology: Problems and Prospects*. New Delhi: SSDN Publishers.

Onyango, R. (2018). Participatory Monitoring and Evaluation: An Overview of Guiding Pedagogical Principles and Implications on Development. *International Journal of Novel Research in Humanity and Social Sciences*, *5*(4), 428-433.

Otieno, J.K.O., Munyua, C.N., & Olubandwa, A. (2016). Effects of Participatory Monitoring and Evaluation on Stakeholder Relationships and Project Quality in the Local Authority Service Delivery Action Planning (LASDAP) Processin Bondo Sub County in Siaya County, Kenya. *Developing Country Studies*, *6*(4), 82-86.

Ransom, H., & Olsson, J.M. (2017). Allocation of Health Care Resources: Principles for Decision-making. *Pediatrics in Review*, *38*(7), 320-329; DOI: https://doi.org/10.1542/pir.2016-0012

Rossman, G. B. (2015). *Participatory Monitoring & Evaluation*. Amherst: The Center for International Education, University of Massachesetts.

Sifunjo, A.N. (2019). *Participatory monitoring and evaluation and successful implementation of maternal health projects within Kajiado north constituency, Kajiado County*, Unpublished MBA Thesis. Nairobi: African Nazarene University.

UNICEF (2020). Kenya: demographics, health, and infant mortality. Available: https://data.unicef.org/country/ken/ [Accessed 8 Oct 2021].

Waweru, E., Opwora, A., Toda, M., Fegan, G., Edwards, T., Goodman, C., & Molyneux, S. (2013). Are Health Facility Management Committees in Kenya ready to implement financial management tasks Findings from a nationally representative survey? *BMC Health Services Research*, *13*, 404. 10.1186/1472-6963-13-404.

World Health Organization, Organisation for Economic Co-operation and Development, and The World Bank. (2018). *Delivering quality health services: a global imperative for universal health coverage*. Geneva: World Health Organization, Organisation for Economic Co-operation and Development, and The World Bank.

APPENDICES

Appendix 1: Participant's Consent Form

Dear Participant,

My name is Pauline Adhiambo Oginga. I am a graduate student at Mount Kenya University. I'm working on a project titled THE INFLUENCE OF PARTICIPATORY MONITORING AND EVALUATION ON DECISION-MAKING IN MATERNAL AND NEWBORN HEALTH (MNH) PROGRAMS IN MOMBASA COUNTY. The outcomes of this study will produce new information in this area and will be used to enhance PM&E processes and decision making in the department of health in Mombasa County and other Kenyan counties. As a consequence, counties, communities, and people will benefit from higher PM&E and decision-making quality.

Procedure to be followed

You will participate in this study by answering the questions on the questionnaire/interview guide given.

Your participation is entirely voluntary. If you opt not to participate in the study, you will not be penalized or prosecuted, and your decision will not be used against you or have an impact on your employment.

You may ask questions about the study at any time. You are free to opt not to answer any questions or to withdraw from the research at any moment.

Discomfort and Risks

If you are uncomfortable answering some questions, you have the option of rejecting to do so. You are also free to terminate the interview at any moment. The interview might go on for up to 60 minutes.

Benefits

You will be aiding us in enhancing the PM&E procedures in the health sector in Mombasa County and Kenya in general by participating in this research. As a consequence, healthcare clients in Mombasa County will benefit from higher levels of healthcare quality. This research is crucial for the advancement of health-care systems since it will provide new information in this area, allowing decision-makers to make evidence-based decisions.

Rewards

Anyone who agrees to participate in the study will not be compensated financially.

Confidentiality

The interview will be held in a private area of the hospital or office. The questionnaires/interview guides will not expose your identify, and the questionnaires/interview guides will be retained in the researcher's secure custody.

Dissemination

The outcomes of the study will be submitted to MKU for the award of a master's degree in public health and published in a reputable health magazine. The findings will also be provided to the Mombasa County Department of Health in order to elicit appropriate measures for improving PM&E and decision making in the county's MNH initiatives.

Contact Information

If you have any questions regarding the study or your participation in it, please call the researcher at 0727-624196 or the Chairman of Mount Kenya University's Institutional Ethical Review Committee (IERC) at P.O. Box 342-01000 or 0720 790 796.

Participant's Statement

The aforementioned information about my involvement in the study is self-evident to me. I was given the chance to ask questions, and my issues were adequately handled. My participation in this study is completely voluntary. I understand that my records will be kept confidential and that I am free to leave the study at any time. I realize that whether or not I discontinue the research, I will not be treated unfairly at work, and my decision will have no impact on how I am treated.

Name of Participant……………………………………Signature…………………………

Date…………………………..

Investigator's Statement

I, the undersigned, have explained to the volunteer the study methodology, as well as the risks and advantages involved, in a way that s/he understands.

Name of interviewer……………………………………Signature………………………

Date…………………………

Appendix 2: Research Questionnaire

Questionnaire Identification Number…………………………

Instructions: Please tick [√] the correct answer in the box provided and write the appropriate answer in some of the questions in the space provided. Your honesty will be highly appreciated.

Section 1: Demographic Information

1. Which level of the health system do you belong to?

   Level 3 [ ] Level 2 [ ] Level 1 [ ]

2. Kindly indicate your occupation in the health facility or community health unit.

   Facility In-Charge [ ] Health Facility Management Committee Member [ ]

   Nurse [ ] Community Health Worker [ ] Maternity Patient [ ]

3. How old are you? ……………………………………
4. What is your gender?

   Male [ ] Female [ ]

5. How long have you been an in-charge, nurse, management committee member, or a CHW in this health facility/community unit?

   Less than 6 months [ ] 6 months-5 years [ ]

   5-10 years [ ] 10 years and above [ ]

6. As a maternity patient, how long have you been seeking MNH services in this facility?
   Less than 6 months [ ] More than 6 months [ ]

7. Are you knowledgeable about Monitoring and Evaluation (M&E)?

Yes [ ] No [ ]

8. If you are trained on M&E, where did you acquire the training?

Pre-service training [ ] On-job training [ ] Seminars/Workshops [ ]

Others specify..

Section 2: Utilization of PM&E in Maternal and Newborn Health Programs

This section seeks to examine the utilization of PM&E approach in maternal and newborn health (MNH) programs in Mombasa County. Please indicate the frequency with which the following principles are adhered to in the design and implementation of MNH programs in Mombasa County using the scale below;

1 – Strongly Disagree 2 – Disagree 3 – Neutral 4 – Agree 5 – Strongly Agree

**No.**	**Activities**	**1**	**2**	**3**	**4**	**5**
9.	Primary stakeholders are considered as active participants in the initiation, design and design, and implementation of MNH programs					
10.	The capacity of primary stakeholders in PM&E (to collect data, analyze, reflect and take action) is enhanced through training and continuous mentorship.					
11.	Joint information reviews and learning among stakeholders is fostered at various levels of PM&E					
12.	Stakeholders are encouraged to be accountable and responsible in taking corrective action(s) based on PM&E information.					

Section 3: PM&E Approaches at the Initiation Phase of MNH Programs

This section seeks to examine the frequency of utilization of selected PM&E approaches at the initiation phase of MNH programs in Mombasa County. Using a 5-point rating scale, please indicate the level of frequency with which the following PM&E approaches are conducted in the initiation of MNH programs. The rating scale is as shown below, whereby 1 is the lowest score while 5 is the highest score;

1 – Not at all 2 – Sometimes 3 – Frequently 4 – Often 5 – Always

No.	Activities	1	2	3	4	5
13.	Participatory needs assessment					
14.	Participatory baseline assessment					
15.	Participatory analysis of objectives					

Section 4: PM&E Approaches at the Design and Planning Phase of MNH Programs

This section seeks to examine the frequency of utilization of selected PM&E approaches at the design and planning phase of MNH programs in Mombasa County. Using a 5-point rating scale, please indicate the level of frequency with which the following PM&E approaches are conducted in the design and planning of MNH programs. The rating scale is as shown below, whereby 1 is the lowest score while 5 is the highest score;

1 – Not at all 2 – Sometimes 3 – Frequently 4 – Often 5 – Always

No.	Activities	1	2	3	4	5
16.	Participatory feasibility analysis					
17.	Participatory SWOT analysis					
18.	Participatory risk assessment					

Section 5: PM&E Approaches at the Implementation Phase of MNH Programs

This section seeks to examine the frequency of utilization of selected PM&E approaches at the implementation phase of MNH programs in Mombasa County. Using a 5-point rating scale, please indicate the level of frequency with which the following PM&E approaches are conducted in the implementation of MNH programs. The rating scale is as shown below, whereby 1 is the lowest score while 5 is the highest score;

1 – Not at all 2 – Sometimes 3 – Frequently 4 – Often 5 – Always

No.	Activities	1	2	3	4	5
19.	Supportive supervision					
20.	Participatory performance reviews					
21.	Participatory desk reviews					

Section 6: Quality of Decision Making in MNH Programs

This section seeks to examine the quality of decision making in MNH programs in Mombasa County. The study has adopted a modified Quality of Decision Making Orientation Scheme (QoDoS) which is a standardized tool for measuring the quality of decision making. Using a 5-point rating scale, please indicate the level of frequency with which the following Quality Decision Making Practices (QDMPs) are practiced in MNH programs. The rating scale is as shown below, whereby 1 is the lowest score while 5 is the highest score;

1 – Not at all 2 – Sometimes 3 – Frequently 4 – Often 5 – Always

No.	Part 1:QDMPs at the Organizational Level					
	**A. Decision Making Approach**	**1**	**2**	**3**	**4**	**5**
22.	The health facility assesses the consequences of its choices.					
23.	The decision-making process at the health institution is open to the public.					

24.	The health facility makes consistent decisions.					
25.	The health facility makes decisions in a systematic manner.					
26.	The expectations of external stakeholders impact the health facility's decision-making.					
27.	In making decisions, the health facility considers the likelihood of success.					
28.	In its decision-making, the health facility quantifies the likelihood of success.					
29.	In making decisions, the health facility is open to considering better alternatives.					
30.	The health facility supports creative decision making.					
31.	In making decisions, the health facility analyzes uncertainty.					
32.	The health facility offers training in the science of decision making.					
33.	As new information becomes available, the health facility reconsiders its decisions.					
**B.**	**C. Decision Making Culture**	**1**	**2**	**3**	**4**	**5**
34.	Slow decision making has resulted in a poor effect for the health facility.					
35.	Because of the health facility's culture, it is unable to make decisions.					
36.	Organizational politics impact decision-making at the health facility.					
37.	The decision-making at the health facility repeats previous errors.					
38.	Individuals' vested interests impact the health facility's decision-making.					
39.	The health facility undervalues difficulties, which has a negative influence on its own decisions.					
40.	The health facility is still working on renovations that should be terminated sooner rather than later.					
41.	Competitors impact the health facility's decision-making.					

	Part 2: QDMPs at the Individual Level					
	**D. Decision Making Competence**	**1**	**2**	**3**	**4**	**5**
42.	My decisions are informed by my knowledge.					
43.	My decision making is consistent					
44.	In my decision-making process, I take into account uncertainties and unknowns.					
45.	In making decisions, I conduct a SWOT analysis.					
46.	As part of my decision-making process, I give contingencies or feasible possibilities.					
47.	My decision-making process is open and transparent.					
48.	I try to comprehend the context of the decision that is being asked of me.					
49.	I try to comprehend the significance of the judgments I make.					
50.	In making decisions, I take a systematic method.					
51.	In my decision-making, I qualify the likelihood of success.					
52.	In my decision-making, I quantify the likelihood of success.					
53.	I am educated in the science of decision making.					
54.	In making decisions, I rely on intuition.					
55.	When it comes to making difficult decisions, my professional expertise comes in handy.					
**E.**	**F. Decision Making Style**	**1**	**2**	**3**	**4**	**5**
56.	Emotions have a role in my decision-making.					
57.	I've had "paralysis by analysis" as a result of my delayed decision-making.					
58.	A decision that was not made had a detrimental impact on me.					
59.	I continue to make the same blunders in my decision-making as in the past.					
60.	Recent or dramatic occurrences have a significant influence on my decision-making.					
61.	My procrastination has had a bad impact.					
62.	By giving weights to my decisions, I might enhance them.					

63.	I overestimate difficulties, which has a negative influence on my decision-making.					
64.	I'm still working on projects that should be terminated sooner rather than later.					
65.	I believe that I could make better quality decisions.					

Appendix 3: Interview Guide

Interview Identification Number...

Date of Interview:	Interviewer:
Sub-County:	Note-taker:
Start time:	Stop time:

Guiding Questions

1. Can you please tell us about your experience on M&E process in this facility?

Probe:

- *Is the process participatory?*
- *Are various stakeholders involved in M&E? (Name the stakeholders involved).*
- *What was your role in the previously conducted M&E?*
- *What was the role of other stakeholders? (Probe as per the stakeholders mentioned above).*
- *How were the stakeholders involved in the M&E process?*
- *What activities were they involved in? (Planning, data collection, data analysis, dissemination of results).*
- *What other activities were conducted?*
- *Were the needs, interests, views and expectations of different stakeholders considered during the M&E process?*
- *In your opinion does the facility conduct Participatory M&E?*

2. What measures has the facility put in place to ensure stakeholders involvement in M&E?

Probe:

- *What are the existing measures in place?*
- *What was the purpose of putting them in place?*
- *Who backed the measures?*
- *Have any evaluations been conducted to determine their effectiveness?*
- *How efficient are they?*

3. What forums/structures exist for stakeholders to be involved in M&E of interventions and service delivery at the facility?

Probe:

- *At the community*
- *Facility – service providers and management level*
- *County/sub-county level – at the department of health*

4. Let us now talk about your past experience on the utilization of PM&E approaches during MNH programs initiation phase.

Probe:

- *Looking at previous and ongoing MNH programs, what is your opinion about the utilization of PM&E at the initiation phase of the programs?*
- *What were some of the PM&E approaches used at the initiation phase?*
- *In your opinion was PM&E utilized optimally at the MNH program initiation phase?*
- *What are the various ways in which the process may be enhanced if PM&E was not used optimally? (Who should be involved, what actions should be undertaken, and why?)*

5. Let us now talk about your past experience on the utilization of PM&E approaches during MNH programs design and planning phase.

Probe:

- *Looking at previous and ongoing MNH programs, what is your opinion about the utilization of PM&E at the design and planning phase of the programs?*
- *What were some of the PM&E approaches used at the design and planning phase?*
- *In your opinion was PM&E utilized optimally at the MNH program design and planning phase?*
- *What are the various ways in which the process may be enhanced if PM&E was not used optimally? (Who should be involved, what actions should be undertaken, and why?)*

6. Let us now talk about your past experience on the utilization of PM&E approaches during MNH programs implementation phase.

Probe:

- *Looking at previous and ongoing MNH programs, what is your opinion about the adoption of PM&E during MNH program implementation phase?*

- *What were some of the PM&E approaches used at the MNH programs implementation phase?*
- *In your opinion was/is PM&E adopted optimally at the MNH programs implementation phase?*
- *What are the various ways in which the process may be enhanced if PM&E was not used optimally? (Who should be involved, what actions should be undertaken, and why?)*

7. What can you say are the key impacts of PM&E adoption on quality of decision making at the facility level and at the county level?

Probe:

- *What parts of PM&E can help improve MNH service delivery?*
- *What do you believe is the most important change that has occurred at the county/sub-county since PM&E was implemented?*
- *Is it true that stakeholders are expressing themselves more than ever before?*
- *What variables contributed to the observed results?*
- *What advantages has the community reaped as a consequence of PM&E?*
- *What are the advantages to the facility?*

8. What are the key lessons you have learnt in the course of implementing PM&E?

9. What are the challenges in adopting PM&E in MNH programs? *(Probe for each set of challenges how they managed it).*

10. How can the department of health in Mombasa County enhance institutionalization of PM&E?

Probe:

- *What structures should be improved or built?*
- *What types of activity may be sustained?*
- *What resources are needed?*

11. What elements of PM&E are useful for scale up?

Probe:

- *Identify the components. (Ask why they say so for each of the topics outlined.)*
- *What aspects should be changed?*
- *What recommendations do you have for changes?*
- *What would you have done differently, in your opinion? Why?*
- *What suggestions do you have for future PM&E implementation?*

Appendix 4: Map of Mombasa County

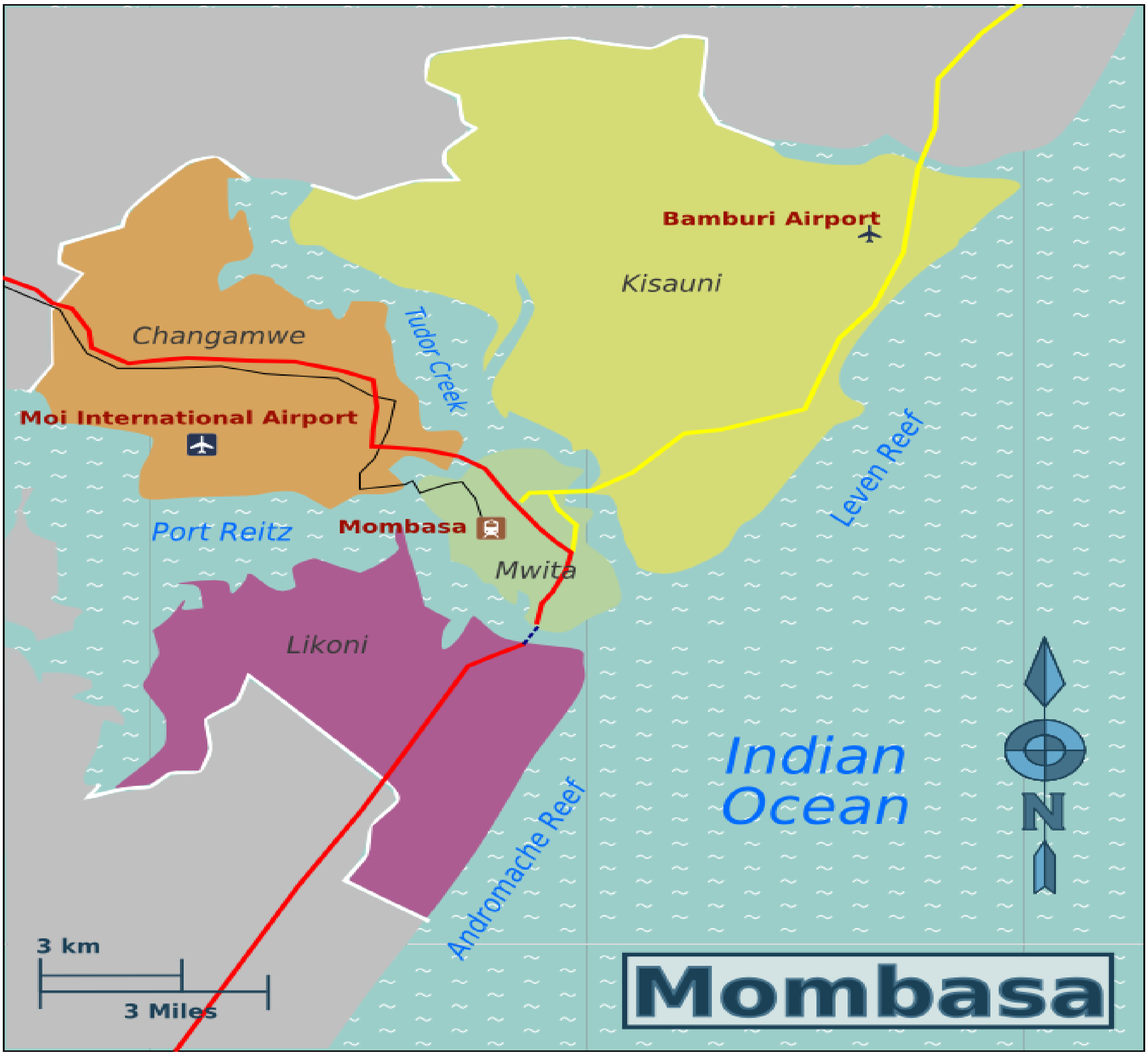

Appendix 5: Distribution of Health Facilities in Mombasa County

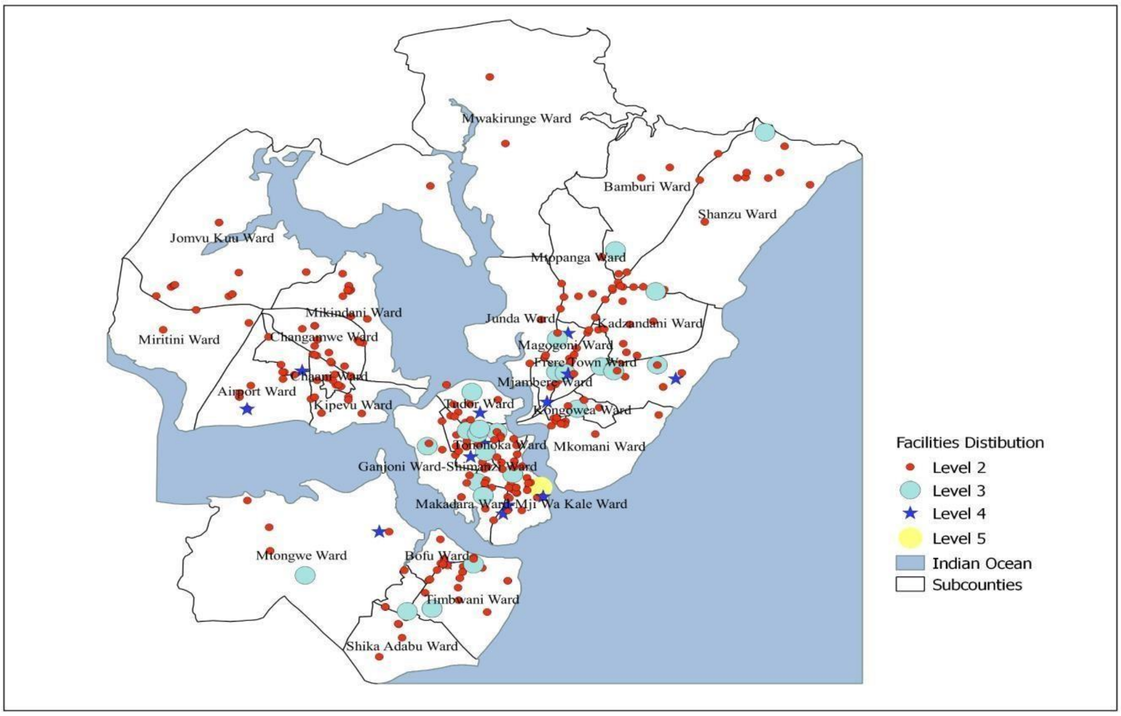

Appendix 6: Ethical Approval

Mount Kenya University

REF: **MKU/ERC/2236**

Date: 17 June 2022

TO: **PAULINE ADHIAMBO OGINGA**

REG: **MPH/2018/39091**

Dear Sir/Madam,

RE: INFLUENCE OF PARTICIPATORY MONITORING AND EVALUATION ON DECISION-MAKING IN MATERNAL AND NEWBORN HEALTH PROGRAMS IN MOMBASA COUNTY, KENYA

This is to inform you that **Mount Kenya University** has reviewed and approved your above research proposal. Your application approval number is **1309.** The approval period is **17/06/2022 - 16/06/2023.**

This approval is subject to compliance with the following requirements;

i. Only approved documents including informed consents, study instruments, MTA will be used
ii. All changes including amendments, deviations and violations are submitted for review and approval by **Mount Kenya University**
iii. Death and life-threatening problems and serious adverse events or unexpected adverse events whether related or unrelated to the study must be reported to **Mount Kenya University** within 72 hours of notification
iv. Any changes, anticipated or otherwise that may increase the risks or affect the safety or welfare of study participants and others or affect the integrity of the research must be reported to **Mount Kenya University** within 72 hours
v. Clearance for export of biological specimens must be obtained from relevant institutions
vi. Submission of a request for renewal of approval at least 60 days prior to expiry of the approval period. Attach a comprehensive progress report to support the renewal
vii. Submission of an executive summary report within 90 days upon completion of the study to **Mount Kenya University**

Prior to commencing your study, you will be expected to obtain a research license from National Commission for Science, Technology and Innovation (NACOSTI) https://research-portal.nacosti.go.ke and also obtain other clearances needed.

Yours sincerely,

The Chairman
Mount Kenya University
Ethics Review Committee
P. O. Box 342 - 0100, Thika

Dr. Peter G. Kirira
Chairman, Mount Kenya University IERC

Main Campus, General Kago Road, P.O. Box 342-01000 Thika.
Tel: 020-2878 000, Cell: +254 709 153 000
[illegible]

Appendix 7: NACOSTI Research Permit

REPUBLIC OF KENYA

NATIONAL COMMISSION FOR SCIENCE,TECHNOLOGY & INNOVATION

Ref No: **963274**

Date of Issue: **21/August/2022**

RESEARCH LICENSE

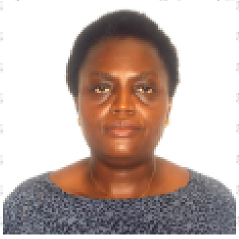

This is to Certify that Ms.. PAULINE ADHIAMBO OGINGA of Mount Kenya University, has been licensed to conduct research in Mombasa on the topic: INFLUENCE OF PARTICIPATORY MONITORING AND EVALUATION ON DECISION MAKING IN MATERNAL AND NEWBORN HEALTH PROGRAMS IN MOMBASA COUNTY, KENYA for the period ending : 21/August/2023.

License No: **NACOSTI/P/22/19461**

963274

Applicant Identification Number

Director General

NATIONAL COMMISSION FOR SCIENCE,TECHNOLOGY & INNOVATION

Verification QR Code

NOTE: This is a computer generated License. To verify the authenticity of this document, Scan the QR Code using QR scanner application.

National Commision for Science, Technology and Innovation -

Appendix 8: Similarity Index Report

Pauline Thesis

by Pauline Odinga

Submission date: 11-May-2023 09:58PM (UTC+0300)
Submission ID: 2090642136
File name: Thesis_PAULINE_ADHIAMBO_OGINGA_Final_11_05_2023_-2.docx (1.61M)
Word count: 23416
Character count: 132861

Pauline Thesis

ORIGINALITY REPORT

13%	12%	4%	3%
SIMILARITY INDEX	INTERNET SOURCES	PUBLICATIONS	STUDENT PAPERS

PRIMARY SOURCES

1	core.ac.uk<br>Internet Source	1%
2	erepository.uonbi.ac.ke<br>Internet Source	<1%
3	www.devolutionhub.or.ke<br>Internet Source	<1%
4	www.strategicjournals.com<br>Internet Source	<1%
5	eprints.lse.ac.uk<br>Internet Source	<1%
6	ir-library.ku.ac.ke<br>Internet Source	<1%
7	www.noveltyjournals.com<br>Internet Source	<1%
8	umispace.umi.ac.ug:8080<br>Internet Source	<1%
9	repository.kemu.ac.ke:8080<br>Internet Source	<1%

Conditional Cash Transfer Project", Journal of Sustainable Development, 2023
Publication

125 Magdalena Bujar, Neil McAuslane, Stuart Walker, Sam Salek. "The Reliability and Relevance of a Quality of Decision Making Instrument, Quality of Decision-Making Orientation Scheme (QoDoS), for Use During the Lifecycle of Medicines", Frontiers in Pharmacology, 2019 <1%
Publication

126 Oguzhan Koyuncu, Hakki Gursoz, Ali Alkan, Hacer Coskun Cetintas, Tuncay Pasaoglu, Emel Mashaki Ceyhan, Stuart Walker. "Evaluation of the Performance of the Turkish Regulatory Agency: Recommendations for Improved Patients' Access to Medicines", Frontiers in Pharmacology, 2020 <1%
Publication

Exclude quotes	Off	Exclude matches	Off
Exclude bibliography	On		
